# High Mg/Ca Molar Ratios Promote Protodolomite Precipitation Induced by the Extreme Halophilic Bacterium *Vibrio harveyi* QPL2

**DOI:** 10.3389/fmicb.2022.821968

**Published:** 2022-04-05

**Authors:** Zuozhen Han, Peilin Qi, Yanyang Zhao, Na Guo, Huaxiao Yan, Maurice E. Tucker, Dan Li, Jiajia Wang, Hui Zhao

**Affiliations:** ^1^Shandong Provincial Key Laboratory of Depositional Mineralization and Sedimentary Minerals, College of Earth Science and Engineering, Shandong University of Science and Technology, Qingdao, China; ^2^Laboratory for Marine Mineral Resources, Qingdao National Laboratory for Marine Science and Technology, Qingdao, China; ^3^Department of Bioengineering, College of Chemical and Biological Engineering, Shandong University of Science and Technology, Qingdao, China; ^4^School of Earth Sciences, University of Bristol, Bristol, United Kingdom; ^5^Cabot Institute, University of Bristol, Bristol, United Kingdom

**Keywords:** extreme halophilic bacteria, protodolomite, Mg/Ca molar ratio, amino acids, polysaccharides, EPS

## Abstract

Bacterial activities have been demonstrated as critical for protodolomite precipitation in specific aqueous conditions, whereas the relationship between the various hydrochemical factors and bacterial activity has not been fully explored. In this study, biomineralization experiments were conducted using a newly isolated extreme halophilic bacterium from salina mud, *Vibrio harveyi* QPL2, under various Mg/Ca molar ratios (0, 3, 6, 10, and 12) and a salinity of 200‰. The mineral phases, elemental composition, morphology, and crystal lattice structure of the precipitates were analyzed by XRD, SEM, and HRTEM, respectively. The organic weight and functional groups in the biominerals were identified by TG-DSC, FTIR, and XPS analysis. The amounts of amino acids and polysaccharides in the EPS of QPL2 cultured at various Mg/Ca molar ratios were quantified by an amino acid analyzer and high-performance liquid chromatography. The results confirm that disordered stoichiometric protodolomite was successfully precipitated through the activities of bacteria in a medium with relatively high Mg/Ca molar ratios (10 and 12) but it was not identified in cultures with lower Mg/Ca molar ratios (0, 3, and 6). That bacterial activity is critical for protodolomite formation as shown by the significant bacterial relicts identified in the precipitated spherulite crystals, including pinhole structures, a mineral coating around cells, and high organic matter content within the crystals. It was also confirmed that the high Mg/Ca molar ratio affects the composition of the organic components in the bacterial EPS, leading to the precipitation of the protodolomite. Specifically, not only the total EPS amount, but also other facilitators including the acidic amino acids (Glu and Asp) and polysaccharides in the EPS, increased significantly under the high Mg/Ca molar ratios. Combined with previous studies, the present findings suggest a clear link between high Mg/Ca molar ratios and the formation of protodolomite through halophilic bacterial activity.

## Introduction

The origin of sedimentary primary dolomite is controversial and has been referred to as one aspect of “the dolomite problem” (Warren, 2000; McKenzie and Vasconcelos, 2009). The “dolomite problem” is based on two facts: (1) dolomite is common in pre-Holocene strata, forming massive dolostone units in the Paleozoic and Precambrian notably, but it is relatively rare in modern marine environments even though seawater is generally oversaturated with respect to dolomite (Burns et al., 2000; Warren, 2000; Gregg et al., 2015) and (2) dolomite has not been synthesized at Earth’s surface conditions (25°C, 1 atm) abiotically (Land, 1998; Braithwaite et al., 2004). During the past century, many attempts have been made to understand this persistent multifaceted problem, and two crucial kinetic obstacles that may impede primary dolomite precipitation have been identified, namely the hydrated nature of magnesium (Lippmann, 1982; de Leeuw and Parker, 2001) and the low activity of ${\text{CO}}_{3}^{2-}$ (Brantley, 2003; Gregg et al., 2015). However, the “dolomite problem” is still an outstanding scientific conundrum and needs to be further investigated.

Mounting evidence confirms that dolomite is more common in modern evaporitic saline environments than it is in modern, open-marine sediments. In the past decades, dolomite has been frequently found in modern saline lakes (Deckker and Last, 1988; Deng et al., 2010; Samylina and Zaytseva, 2019; Li et al., 2020), coastal evaporitic sabkhas (Bontognali et al., 2010; Shalev et al., 2020; Diloreto et al., 2021), and saline soils (Sherman et al., 1947; Kohut et al., 1995), while by contrast, almost no dolomite precipitation has been reported in the ocean (Gregg et al., 2015). The concentrations of various ions in these evaporative saline systems are significantly higher than those in surface seawater, including Na^+^, Ca^2+^, Mg^2+^, Cl^–^, and ${\text{SO}}_{4}^{2-}$ (Deng et al., 2010; Kolpakova et al., 2019), leading to the inference that dolomite precipitation may be linked to high salinity, high sulfate concentration, and increasing Mg/Ca molar ratios. However, abiotic laboratory experiments show that the conditions of supersaturation with respect to dolomite are insufficient to overcome the kinetic obstacles for dolomite precipitation under the normal temperature and pressure of the Earth’s surface (25°C, 1 atm).

An increasing number of studies suggest that microbial activities may play a key role in the precipitation of dolomite. Evidence from ancient dolostones, including microbial fossils (Sun et al., 2020), various bacterial organic signatures (Perri and Tucker, 2007; Baldermann et al., 2015), and a complex mineral morphology (You et al., 2013) has led to the consideration of their bacterial origin or at least a microbial involvement for some types of dolomite. In addition, laboratory culture experiments have further confirmed that dolomite can be induced by various bacteria. To date, at least four types of bacteria have been implicated for their ability to induce dolomite precipitation, including halophiles (Sánchez-Román et al., 2009; Qiu et al., 2017), sulfate reducing bacteria (SRB) (Bontognali et al., 2014), methanogenic archaea (Zhang et al., 2021), and planktic aerobic heterotrophic bacteria (Liu et al., 2019). Among these, halophilic bacteria are a unique type that only live in highly saline environments (Oren, 2008). It has been demonstrated that halophiles can modify ion concentrations in the surrounding microenvironment so that dolomite reaches saturation near the cell (Sánchez-Román et al., 2009). Not only that, but the cell wall and extracellular polymeric substances (EPS) of halophilic bacteria can provide nucleation sites for dolomite precipitation by adsorbing cations as a result of the presence of negatively-charged organic functional groups (Sánchez-Román et al., 2009; Qiu et al., 2017). Halophilic and halotolerant bacteria are abundant microorganisms in hypersaline environments (Sánchez-Román et al., 2009; Al Disi et al., 2017; Qiu et al., 2017), including the chemo-organotrophic halophilic bacteria (Jiang et al., 2006; Al Disi et al., 2017), archaea (Borsodi et al., 2013), and SRB (Foti et al., 2007; Jiang et al., 2007). The precipitation of dolomite in the above-mentioned saline environments is essentially related to these bacterial activities (Vasconcelos et al., 1995; Qiu et al., 2017).

The precipitation of bacterially-induced dolomite is usually associated with and restricted by particular conditions, especially the solution temperature, the sulfate concentration, salinity, and increasing Mg/Ca molar ratio (Sánchez-Román et al., 2011; Qiu et al., 2017; Al Disi et al., 2019; Liu et al., 2019). For example, protodolomite has been induced by *Haloferax volcanii* DS52 in a medium with salinity (NaCl) higher than 200‰ (Qiu et al., 2017), whereas aragonite and monohydrocalcite were formed in other conditions. The interactions between bacteria and the above-mentioned ambient factors have been examined in previous studies. (1) A higher solution temperature is certainly favorable for dolomite precipitation (e.g., Al Disi et al., 2019), leading to increases in MgCO_3_ and cation ordering in both abiotic and the biotic experiments (Malone et al., 1996; Al Disi et al., 2019), since elevated temperature increases the saturation index of carbonate minerals (Arvidson and Mackenzie, 1999). A higher temperature is also beneficial for overcoming kinetic obstacles to precipitation (Rodriguez-Blanco et al., 2015). (2) The presence of sulfate does not inhibit low-temperature bacterially-induced dolomite precipitation in natural environments (e.g., Vasconcelos and McKenzie, 1997) or under laboratory conditions (Sánchez-Román et al., 2009), contrary to earlier indications (Baker and Kastner, 1981). (3) High salinity facilitates dolomite precipitation mediated by halophilic bacteria (Qiu et al., 2017; Al Disi et al., 2019). Experiments have shown that an increase in salinity enhances the amount of carboxylated molecules on the cell surface (mainly the acidic amino acids), leading to the nucleation of protodolomite (Qiu et al., 2017). (4) Many studies have suggested that an increasing Mg/Ca molar ratio will enhance the saturation index of dolomite, although experimentally dolomite does not appear to form in solutions with a Mg/Ca ratio less than 9 (Land, 1998). Many bacterially-induced dolomites have been linked to high Mg/Ca molar ratio conditions, e.g., Mg/Ca = 10 (Qiu et al., 2017), Mg/Ca = 9.7 and 18 (Bontognali et al., 2012), Mg/Ca = 7.5 (Sánchez-Román et al., 2009, 2011), and Mg/Ca = 15 (Liu et al., 2019). It has also been found that other Mg-carbonate minerals can be induced under high Mg/Ca conditions, e.g., Mg/Ca = 12 (Zhao et al., 2019, 2021) and Mg/Ca = 9 (Pan et al., 2019). Taken together, the combination of higher Mg/Ca ratios and bacterial activity may promote the precipitation of dolomite, although this has not yet been investigated in detail.

To explore the interaction between variations in Ma/Ca molar ratios and bacterially-induced precipitation, a newly isolated extreme halophilic bacterium, *Vibrio harveyi* QPL2, was cultured in a medium with 200‰ salinity and various Mg/Ca molar ratios. From these experiments, the biominerals precipitated were characterized for their mineralogy, crystal structure, and chemistry and analyses were undertaken of the organic composition of bacterial EPS. Our results shed new insights into the mechanism of halophilic bacterially-induced protodolomite formation.

## Materials and Methods

### Isolation, Identification, and Culture of the Halophilic Strain

The sample from which halophilic bacteria were isolated was collected from salina mud at Yinjiashan Salt Farm (Qingdao, China) (35.6598°N, 119.8414°E). The sediments are alternating dark and light laminae (Supplementary Figure 1) and a sample from the dark layer was dissolved in an enriched Luria-Bertani Medium (LB) with 200‰ salinity. After 48 h of incubation in a constant temperature incubator (30°C), the enriched medium became significantly turbid, indicating that the halophilic bacteria in the medium had begun to multiply. Twenty microliters of the above turbid medium were evenly spread on the solid LB medium, and after 48 h of constant temperature incubation, a dominate single colony was then inoculated into the LB liquid medium. A purified halophilic bacterium named QPL2 was obtained after repeating the inoculation and isolation process three times. The purified bacterial colonies were identified by 16S ribosomal deoxyribonucleotide (16S rDNA) at Shanghai Sangon Biotech Co., Ltd. (Shanghai, China). The complete 16S rDNA sequence was uploaded to GenBank of the National Center for Biotechnology Information (NCBI) and the BLAST program was applied to obtain the results of the 16S rDNA homology. A multiple sequence alignment was erected with the ClustalX software (version 2.0), and a phylogenetic tree of QPL2 was constructed with MEGA 6.0 using the neighbor-joining method. A series of physiological and biochemical experiments including optimal salt concentration, ammonia production, and carbonic anhydrase activity were also conducted to further confirm the taxonomy of QPL2.

### Biomineralization Experiments

#### The Conditions of the Bioreactors

The medium used for the biomineralization experiments was LB medium with a salinity of 200‰ and additional Ca^2+^ and Mg^2+^ ions (Supplementary Table 1). All of the inorganic reagents (KCl, NaCl, CaCl_2_, and MgCl_2_⋅6H_2_O) are of analytical grade. The beef extract, tryptone, is biotech grade. All were purchased from Sinopharm Chemical Reagent Co., Ltd. (Beijing, China) and used as received. Deionized water was used in all experiments. Stock solutions of CaCl_2_ and MgCl_2_⋅6H_2_O were added into the LB medium to adjust the Mg/Ca molar ratios. The final Ca^2+^ concentration was fixed to be 0.01 M, while the Mg/Ca molar ratio was adjusted to be 0, 3, 6, 10, and 12, respectively. The initial pH of the medium was adjusted to 6.8 using HCl solution (1%) and NaOH solution (1%). About 5 mL of the cell suspension from the bacterial enrichment medium in the stable growth phase was inoculated in 150 mL LB medium in a vertical flow clean bench. All of the groups were made in triplicates and were cultured at 37°C with constant rotation at 130 rpm. In the biomineralization experiments, the medium solutions were periodically sampled to determine the pH values, concentrations of CO_3_^2–^, HCO_3_^–^ and ammonia according to the published methods (Zhuang et al., 2018; Zhao et al., 2021).

#### Preparation of the Abiotic Experiments

Experiment 1: non-inoculated medium with an initial pH of 6.8. The components of the medium were the same as those of the bioreactor (LB medium with a salinity of 200‰), and the pH value was adjusted to 7.0. The Mg/Ca molar ratio of the medium was also adjusted by CaCl_2_ and MgCl_2_⋅6H_2_O stock solution to be 0, 3, 6, 10, and 12, respectively.

Experiment 2: non-inoculated medium with an initial pH of 8.5. The components and Mg/Ca molar ratios of the medium were the same as those of experiment 1, and the pH value was adjusted using NaCO_3_ solution (0.5 M).

Experiment 3: Synthesis of protodolomite in inorganic solution at 60°C. Protodolomite was prepared by sol-gel and hydrothermal methods for comparison with biotic protodolomite (Malone et al., 1996; Zheng et al., 2021). Stock solutions of CaCl_2_ (1 M), MgCl_2_⋅6H_2_O (1 M) and Na_2_CO_3_ (1 M) were added into a flask with the volume ratio of 1:1:2. The obtained sol-gel solution was then placed in an oven at 60°C for 48 h, and the minerals were washed, precipitated, and naturally dried for later use.

All of these experiments were also made in triplicate and were cultured at 37°C with constant rotation at 130 rpm.

### Mineral Characterization

After 15 days of culture, the precipitates in the medium were taken out for analysis. The precipitates were collected from the bottom of the flask after 10 mins of ultrasonic vibration and 5 mins of standing. Before various analyses, the precipitates were washed with deionized water three times to remove any medium mixed with the particles.

The mineralogy of the precipitates was analyzed by X-ray diffraction (D/Max-RC, Rigaku Co., Tokyo, Japan) with Cu-Kα radiation, a scanning angle (2θ) range from 10°to 60°, a scanning speed of 8° min^–1^, and a step size of 0.02°. The XRD data were processed by MDI Jade 6.5 software.

The morphology and elemental composition of the precipitates were determined by scanning electron microscopy (SEM, Hitachi S-4800, Japan) with an X-ray energy dispersive spectrometer (EDS, Apollo XLT SDD, United States). The electron accelerating voltages of SEM and EDS were 5 and 10 kV, respectively.

The detailed crystal structures of the precipitates were measured by high-resolution transmission electron microscopy and selected area electron diffraction (HRTEM-SAED, FEI TalosF200S, United States) at an electron accelerating voltage of 200 kV.

The weight percentage of organics in the minerals was analyzed by thermogravimetry and differential scanning calorimetry (TG-DSC, STA449F5, Germany) from 25 to 1,000°C at a heating rate of 15°C⋅min^–1^. The organics in minerals were also analyzed by Fourier-transform infrared spectroscopy (FTIR, Nicolet 380, Thermo Fisher Scientific Inc., MA, United States) with the wavenumber ranging from 4,500 to 500 cm^–1^; they were also analyzed by X-ray photoelectron spectroscopy (XPS, Thermo ESCALAB 250XI; Axis Ultra DLD Kratos AXIS SUPRA; PHI-5000versaprobe) with the binding energy from 1,300 to 0 eV. The minerals induced by the bacteria were also analyzed by Raman spectroscopy at 532 nm (DXR2, Thermo Fisher Scientific Inc., Waltham, MA, United States).

### Extraction of Extracellular Polymeric Substances

The EPS of the bacteria were extracted by the heating-dialysis method (Morgan et al., 1990). The EPS were removed from the medium of the seed liquid (medium without Ca^2+^ and Mg^2+^), and the medium with Mg/Ca molar ratios of 0, 3, 6, 10, and 12 at 100 h after inoculation. A blank un-inoculated medium was used as a control. The cell aggregates were collected from 150 mL medium by centrifugation at a speed of 3,000 rpm for 10 mins; after that, the cell aggregates were drawn out by pipette and re-suspended in 50 ml of NaCl solution with a salinity of 200‰. The bacteria were then centrifuged at 2,000 rpm for 3 mins to remove any medium components, and this was repeated three times. The suspended cells were incubated in a water bath at a temperature of 37°C for 30 mins, followed by centrifugation at a speed of 10,000 rpm for 10 mins. The supernatant was filtered through a microporous membrane (0.22 μm) to further remove any residual cells entirely. The transparent liquid was the solution of EPS and NaCl. To remove the NaCl, the solution was transferred to dialysis bags (DM = 25 mm, MwCO = 500 Da) and dialyzed against deionized water for 48 h to remove the ions. After that, the solution inside the dialysis bag was transferred to centrifuge tubes and vacuum freeze-dried at a temperature of −60°C. The EPS powder obtained was stored in a refrigerator at −20°C for later analysis.

The amino acids in the EPS were examined by an amino acid analyzer (L-8900, Hitachi, Japan). The monosaccharide components in EPS were detected by High Performance Liquid Chromatography (LC-20AD, Shimadzu, Japan). The samples were treated by derivative acidolysis and the data were calculated by the single point area normalization method. Twelve monosaccharide components, including mannose, glucose, galactose, glucuronic acid, and galacturonic acid, were tested. Since protein, polysaccharide, and uronic acid are the main components of EPS, the contents of these three substances in EPS were determined (Bazaka et al., 2011). The protein content was determined using a Bradford Protein Assay Kit. The polysaccharide content in EPS was determined by the phenol-sulfuric acid method with glucose as the standard. The content of uronic acid was determined by the hydroxydiphenyl method with glucose as a standard.

## Results

### Identification of the Strain

**FIGURE 1:**
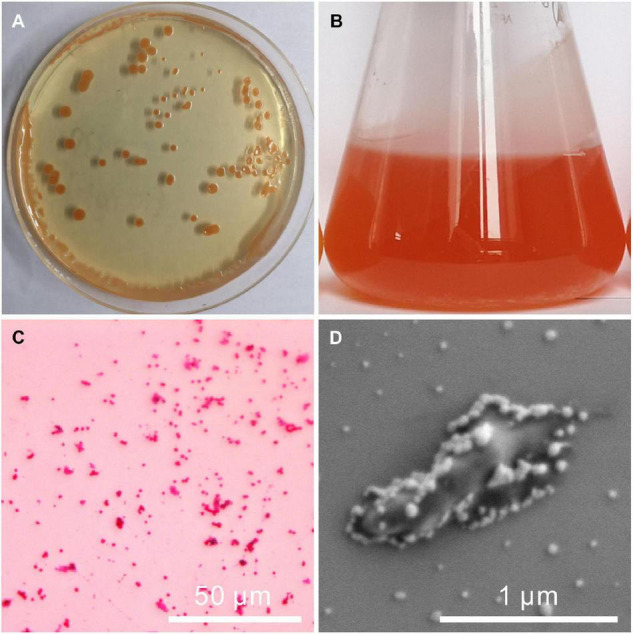
Images of the colony, seed liquid, and a single cell of *Vibrio harveyi* QPL2. **(A)** Colonies of QPL2 on a solid medium; **(B)** culture medium of QPL2 showing orange color and bacterially-induced precipitates; **(C)** negative gram-staining results; and **(D)** single cell of QPL2.

The fully developed single colony of the strain QPL2 grown on the solid LB medium at 37°C for 100 h was moist, circular in shape with an entire margin, and was light orange in color (Figure 1A). When the strain was incubated in the liquid LB medium, the solution showed a tangerine color (Figure 1B). The appearance of this color may be related to the properties of halophilic bacteria that can produce carotenoids (de Lourdes Moreno et al., 2012), and is similar to the color of many salt lakes in the natural environment (Baxter, 2018). The red staining results of QPL2 confirmed that the strain is Gram-negative bacteria (Figure 1C). The strain also showed positive results for ammonia production (Supplementary Figure 2). The single cell of QPL2 is about 1 μm in length and 0.3 μm in width (Figure 1D). The cell is coated with viscous EPS (Figure 1D). The small particles on the surface of the cell are crystalline NaCl.

**FIGURE 2:**
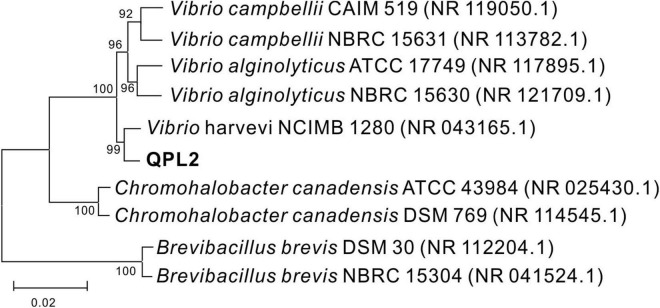
Phylogenetic tree of *Vibrio harveyi* QPL2 and its closest relatives based on the 16S rDNA sequence, which was constructed by the neighbor-joining method.

The 16S rDNA sequence of QPL2 bacteria was detected to be 1,451 base pairs (bp) in the length. The complete 16S rDNA sequence was uploaded to the GenBank of the NCBI, and received an accession number of OK493384. According to BLAST results, QPL2 bacteria shared 99% homology with the bacteria belonging to *Vibrio harveyi*. The phylogenetic tree of *Vibrio harveyi* QPL2 bacteria was constructed by the neighbor-joining (NJ) method using MEGA 6.0 software; the result shows that the strain was clustered with members of *Vibrio harveyi* strains (Figure 2). Based on these results, the present strain was identified as *Vibrio harveyi* QPL2.

### Growth and Hydrochemistry in the Medium

**FIGURE 3:**
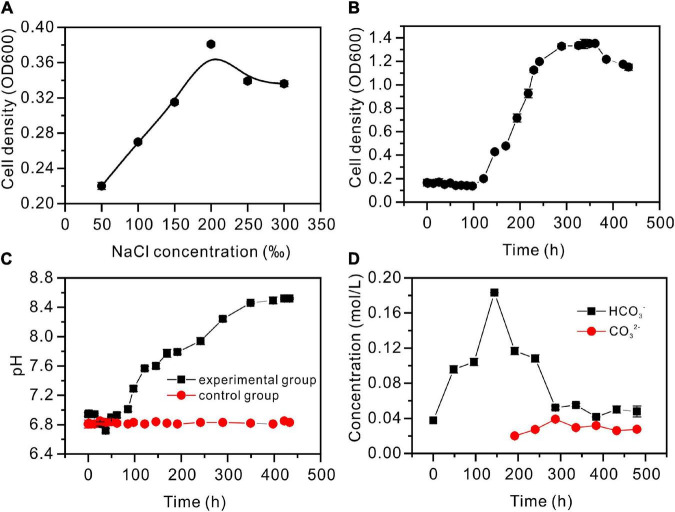
Hydrochemical characteristics of the *Vibrio harveyi* QPL2 seed liquid without the addition of Ca^2+^ and Mg^2+^. **(A)** Salinity adaptation experiment; **(B)** cell density growth curve through time; **(C)** pH curves through time; and **(D)** the changes in HCO_3_^–^ and CO_3_^2–^ concentrations through time.

The strain *Vibrio harveyi* QPL2 was cultured under various salinities (50, 100, 150, 200, 250, and 300‰). The results show that QPL2 reached the highest cell density (OD_600_ = 0.381) when the salinity was 200‰, after cultured 100 h (Figure 3A). Moderate (<150‰) or excessive (≥250‰) salinity is not conducive to the survival of QPL2 (Figure 3A). This confirms that it is an extreme halophilic bacterium. The detailed growth curve of QPL2 in a medium with 200‰ salinity is shown in Figure 3B, and it can be divided into four stages: (1) In the delay phase (0–121 h), the OD_600_ value is almost always between 0 and 0.1; (2) In the exponential growth stage (121–289 h), the OD_600_ quickly rose to the highest value (about 1.32); (3) In the stable stage (289–362 h), the OD_600_ value is stable at the highest peak; (4) During the decline stage (362–434 h), due to the consumption of nutrients in the medium, the number of cells tends to decrease.

The pH values are shown in Figure 3C, with significant differences between the experimental and control groups. For the first 13 h, the experimental groups were almost unchanged. As a result of the hydration of CO_2_, pH decreased during 13–17 h; pH then gradually increased, to remain almost unchanged at 8.52. As shown in Supplementary Figure 2B, CA activity increased slowly before 72 h, rose sharply to 14 (U/mL) between 72 and 264 h, and then gradually decreased. Since CA can catalyze the hydration of CO_2_ to produce ${\mathrm{HCO}}_{3}^{-}$ and ${\text{CO}}_{3}^{2-}$, ${\text{HCO}}_{3}^{-}$ and ${\text{CO}}_{3}^{2-}$ values were determined (Figure 3D). The ${\mathrm{HCO}}_{3}^{-}$ concentration increased sharply, reaching the maximum at 144 h, and then decreased gradually. However, it was not until 192 h that ${\text{CO}}_{3}^{2-}$ was produced. The curve of NH_4_ ion concentration is shown in Supplementary Figure 2A. In the exponential growth stage of bacteria, the concentration of NH_4_ reaches the highest value (3.2 × 10^–5^ mol/L). These processes contribute to the alkaline nature of the medium.

### Mineralogy of Precipitates Induced by *Vibrio harveyi* QPL2

**FIGURE 4:**
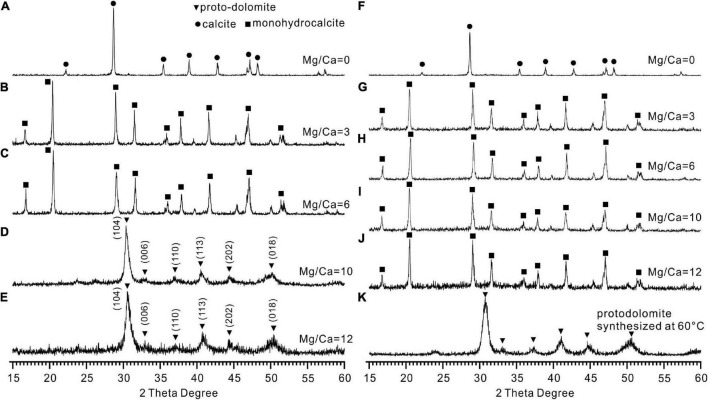
X-ray diffraction (XRD) patterns of the minerals induced by *Vibrio harveyi* QPL2 and the precipitates in the abiotic control experiments. **(A–E)** Minerals induced by QPL2 in the mediums with Mg/Ca molar ratios of 0, 3, 6, 10, and 12. **(F–J)** Minerals in the abiotic experiments with Mg/Ca molar ratios of 0, 3, 6, 10, and 12. **(K)** Protodolomite synthesized in pure inorganic solution at 60°C.

The XRD results revealed that calcite (CaCO_3_), monohydrocalcite (CaCO_3_⋅H_2_O), and protodolomite were all induced by QPL2 in the biotic experiments. The crystals formed in the absence of Mg^2+^ were exclusively calcite (CaCO_3_) (Figure 4A). Monohydrocalcite (CaCO_3_⋅H_2_O) was the sole component of the precipitates formed in the biotic reactor with the Mg/Ca molar ratios of 3 and 6 (Figures 4B,C). When the Mg/Ca molar ratio rises further, the mineralogy of the solid products changes significantly. It can easily be observed that the spectra of the precipitates are less precise compared to those of calcite and monohydrocalcite, indicating that the crystallinity of the minerals is lower (Figures 4D,E). The wider full width at half maximum (FWHM) of the highest peak (1.93°) compared to calcite (0.62°) and monohydrocalcite (0.65°) also confirms this difference (Figures 4D,E).

**FIGURE 5:**
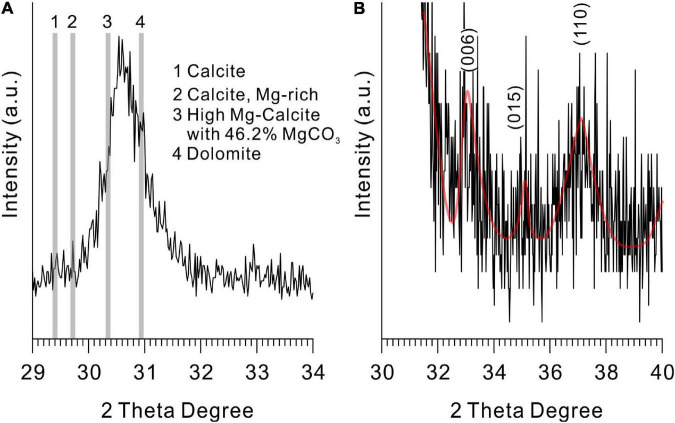
X-ray diffraction spectra of protodolomite induced by QPL2 at a Mg/Ca molar ratio of 12. **(A)** The 2θ position of the (104) face of protodolomite induced by QPL2 and comparison with other Mg-containing carbonate minerals and **(B)** simulated (006), (015), and (110) faces of protodolomite induced by QPL2. The data for standard calcite, Mg-rich calcite, and dolomite are from the MDI Jade 6.5 database [PDF#47-1743, PDF#43-0697, and PDF#36-0426); the data for high-magnesium calcite are from Zhang et al. (2010)].

There is no standard mineral PDF card that fits accurately with the XRD pattern of the precipitates from the medium with Mg/Ca molar ratios of 10 and 12, but it does look very close to protodolomite or dolomite in the literature (Sánchez-Román et al., 2009; Qiu et al., 2017; Liu et al., 2020). Protodolomite is one of the isomorphisms of calcite, but with the mole percentage of MgCO_3_ ranging from 36 to 55% (Zhang et al., 2015). It is well known that the 2θ position or the interplanar spacing (*d* value) is approximately linearly related to the mole percentage of MgCO_3_ (Goldsmith and Graf, 1958). The data presented in Figure 5A show that the (104) 2θ position of the biomineral is shifted to a higher value than standard calcite, Mg-rich calcite, or high Mg-calcite (with 46.2% MgCO_3_). It is reasonable to conclude that the mole percentage of MgCO_3_ in the present biominerals is more than 46.2%. Protodolomite is distinguished from dolomite by lower symmetry and no cation ordering (Gregg et al., 2015). The (101), (015), and (021) are the ordering reflections of ideal dolomite. Even though there is no visible (101) or (015) peak in the pattern, an indistinct (015) peak in the enlarged area seems to be hidden in the background noise (Figure 5B). Thus, the biominerals induced by QPL2 in the medium with Mg/Ca molar ratios of 10 and 12 were identified as protodolomite (Figures 4D,E).

In the abiotic experiments, no minerals formed in the experiment 1 with the initial pH of 6.8. In experiment 2 with the initial pH of 8.5, the abiotic minerals formed in the absence of Mg^2+^ were exclusively calcite (Figure 4F). Monohydrocalcite was always the only precipitate formed in the abiotic experiments with the Mg/Ca molar ratios of 3, 6, 10, and 12 (Figures 4G–J). It is clear that the significant difference between the biotic and abiotic experiments is that the bacterial activities lead to the formation of protodolomite. Protodolomite was also synthesized in purely inorganic solutions at 60°C (Figure 4K).

### Morphology, Elements, and Atomic Arrangement of Microbial Minerals

**FIGURE 6:**
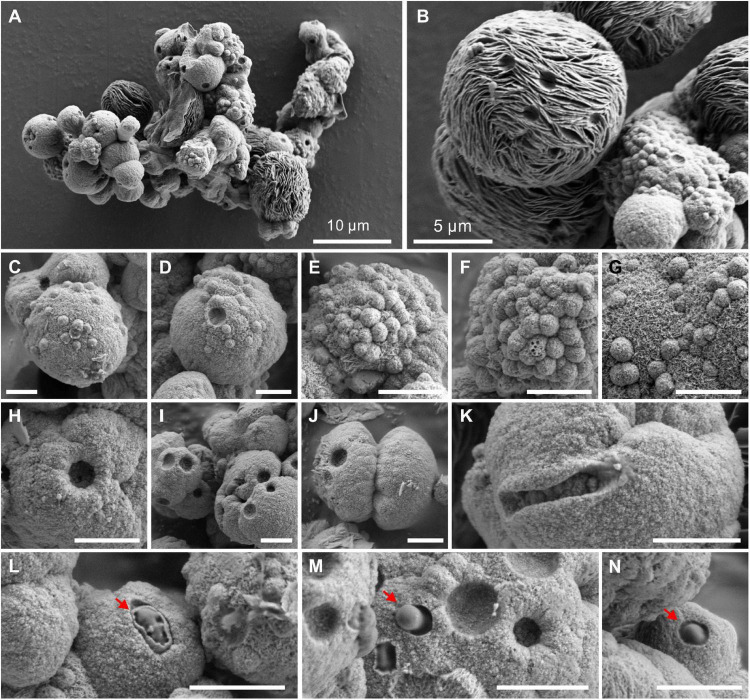
Morphologies of protodolomite induced by *Vibrio harveyi* QPL2 in LB media with a Mg/Ca molar ratio of 12. **(A)** Protodolomite mineral aggregates. **(B)** A spheroidal huntite crystal with a fibrous and flakey structure. **(C–G)** Numerous spheroids with a diameter of about 0.5–1.0 μm on the surface of protodolomite. **(H–K)** Pinholes with a diameter of approximately 1 μm on the knobby surfaces of protodolomite. **(L–N)** Living bacteria enveloped within the mineral crust. The scale bar of the images **(C–N)** is 2 μm.

The biotic protodolomite crystals formed in a medium with the Mg/Ca molar ratio of 12 are usually aggregations of single and double spheres (Figure 6A). There are also spheres with flakey surface patterns (Figure 6B), which are similar to the huntite [CaMg_3_(CO_3_)_4_] reported in the experiments of Sánchez-Román et al. (2011). However, our XRD data do not show the diffraction peak of huntite, which may be due to its low content and poor crystallinity. The detailed surface morphology of the biotic protodolomite is shown in Figures 6C–N. The surface of the biotic protodolomite is always rough and loose with a knot-like structure. No smooth crystal planes were observed, which corresponds to its low crystallinity as deduced from the XRD data. This could indicate that the growth speed of these minerals was very fast. It is interesting to note that there are many micro-globules with a diameter of about 300 nm (Figures 6C–G) on the surface of the biotic protodolomite spheres. These micro-globules have the same surface detail compared to the biotic protodolomite crystals, leading to the possibility that these micro-globules may be the initial growth stage of the biotic protodolomite. One or more pinhole structures are observed on the surface of almost every sphere (Figures 6H–K); similar features are reported by Qiu et al. (2017). These pinholes could be produced where micro-globules have fallen out. However, we suggest they are more likely to be a relic feature of where the growth of protodolomite has taken place around a bacterial cell, since the pinhole has penetrated the crystal (Figure 6K), and the diameter and size of the pinholes are very close to that of the QPL2 cell. At the same time, bacteria with a mineral envelope (Figure 6L, marked by red arrow) and living bacteria embedded in the mineral surface (Figures 6M,N, marked by red arrow) can be clearly recognized. These features suggest that there is a close relationship between the formation of protodolomite and bacterial activity.

**FIGURE 7:**
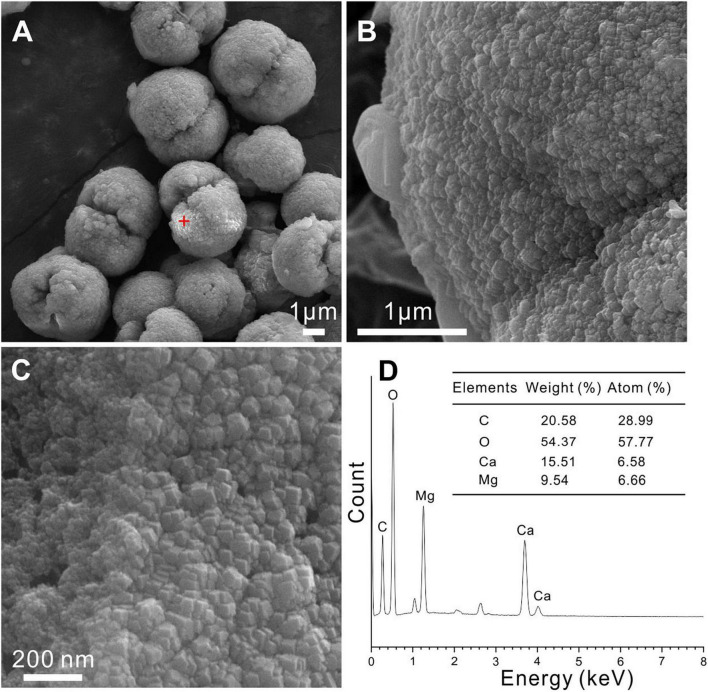
Protodolomite synthesized in a purely inorganic solution at 60°C. **(A–C)** The morphology and surface patterns of the abiotic protodolomite. **(D)** EDS analysis of the selected spot in panel **(A)**.

However, protodolomite precipitates in the form of spherulites and dumbbells have also been reported from purely abiotic experiments (Zheng et al., 2021). To compare the morphology of the biotic protodolomite and that of the abiotic protodolomite, we synthesized protodolomite in pure abiotic solutions at 60°C according to published methods (Malone et al., 1996; Zheng et al., 2021). Basically, each abiotic protodolomite crystal shows a similar dumbbell shape with a diameter about 4 μm (Figure 7A), whereas the single crystal size and morphology of biotic protodolomite show a range (Figure 6A). The enlarged view of abiotic protodolomite shows that the crystals are composed of numerous subunits, but these subunits are near-euhedral rhombohedra (Figures 7B–D) rather than having the micro-globular morphology of biotic protodolomite. Through the above comparison, it can be seen that there are significant differences in morphology between “low-temperature biotic protodolomite” and “high-temperature protodolomite” synthesized in an inorganic solution; which further indicates that the bacteria play an important role in the precipitation of protodolomite.

**FIGURE 8:**
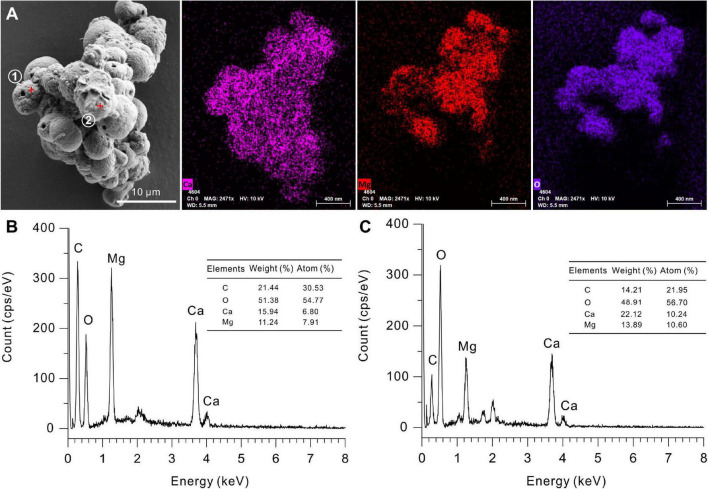
Elemental composition of protodolomite induced by *Vibrio harveyi* QPL2 in LB media with different Mg/Ca molar ratios. **(A)** Elemental mapping of protodolomite aggregates. **(B,C)** Spot EDS analyses of two selected positions in panel **(A)**.

The EDS instrument is a key tool to determine the elemental composition of protodolomite (Zhang et al., 2010). The element maps of O, Ca, and Mg from biotic protodolomite in a medium with a Mg/Ca molar ratio of 12 show a similar distribution (Figure 8A); where the homogenous distribution of Mg and O in the lower right corner may be caused by the excessive change in the height of the sample at this position. About 12 protodolomite spheres were selected for EDS spot analysis. The results of two typical spots from the sample (Figure 8A) are shown in Figures 8B,C. It confirms that biotic protodolomite contains approximately similar contents of Mg and Ca. The content of Mg is slightly more than that of Ca. Based on the atomic weight, there are about 53.8 and 50.8% mole percent MgCO_3_ in the two spots. Therefore, the chemical formulae of the biotic protodolomite in the two spheres are Ca_0.924_Mg_1.076_(CO_3_)_2_ and Ca_0.984_Mg_1.016_(CO_3_)_2_. These results conform with the definition of protodolomite (Zhang et al., 2015).

**FIGURE 9:**
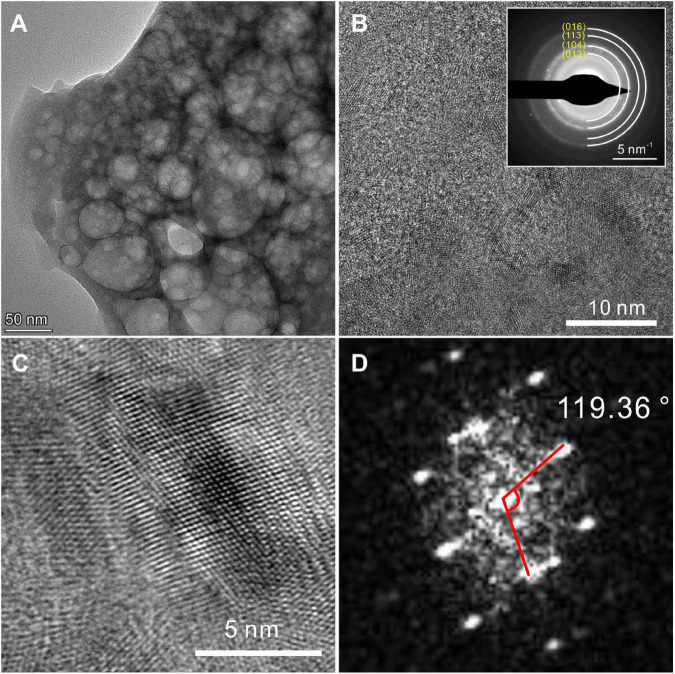
TEM images of protodolomite induced by *Vibrio harveyi* QPL2. **(A)** A bright-field image of dolomite particles. **(B)** The SAED pattern shows diffraction rings of dolomite polycrystals and does not show any super-lattice structure. **(C,D)** The high-resolution image of a selected area and its FFT pattern.

The HRTEM results display the lattice structures of the biotic protodolomite formed in the medium with a Mg/Ca molar ratio of 12. For a polycrystalline area (Figures 9A,B), the SAED pattern shows several diffraction rings including (012), (104), (113), and (016). In addition, no ordering structure is observed, which corresponds to the results of the XRD. For a single crystal area (Figure 9C), where the crystal was analyzed nearly parallel to the “c” axis, the average interatomic distances of the “a” axis and “b” axis are nearly equal, and the angle γ of nearly 120° corresponds to ${\text{R}}_{3}^{-}\,\text{c}$ of protodolomite (Figure 9D).

The morphology and elemental information of the microbial precipitates harvested from the medium with Mg/Ca molar ratios of 0, 3, 6, and 10 are shown in Supplementary Figure 2. The calcite shapes are mainly dumbbells and spheres, the monohydrocalcite mostly twisted. The minerals in the medium with a Mg/Ca ratio of 10 have their spherical surfaces covered in micro-globules.

### Organics in the Precipitates Induced by *Vibrio harveyi* QPL2

#### Weight of Organics in the Protodolomite

**FIGURE 10:**
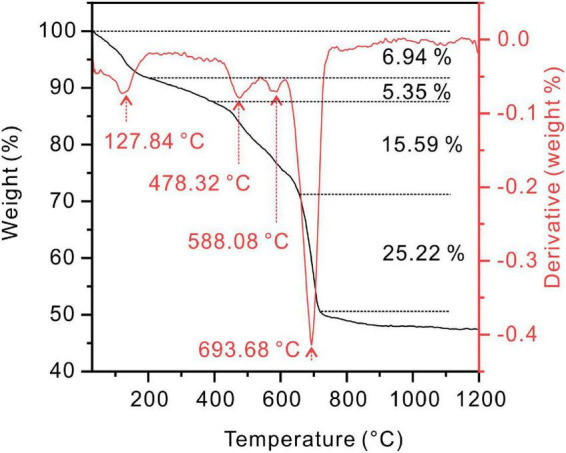
Thermogravimetry and differential scanning calorimetry (TG-DTG) curves of protodolomite induced by *Vibrio harveyi* QPL2 in a medium with a Mg/Ca molar ratio of 10.

The protodolomite induced by the QPL2 in the medium with the Mg/Ca molar ratio of 10 was analyzed for its thermal behavior. From the TG-DTG curve, four stages of mass loss can be recognized (Figure 10). The first weight loss (5.5%) stage was from 30°C to about 128°C. With this temperature being higher than the boiling point of water (100°C), the result indicates that there is not only absorbed water but also bound water in the protodolomite (Hatakeyama et al., 1988; Zheng et al., 2021). The second stage was a 5.3% weight loss from 128 to 478°C; which would correspond to the decomposition of organics in the protodolomite. The third stage with a 15.6% weight loss from 478 to 588°C, can be ascribed to the breakdown of MgCO_3_ into MgO and CO_2_. The last stage of a 21.6% weight loss from 588 to 694°C, is interpreted as the decomposition of CaCO_3_ into CaO and CO_2_. It can be concluded that there is significant organic material within the protodolomite; and these organics probably consist of bacterial cells enclosed within the minerals and molecular organic matter dispersed in the crystal lattice structure.

#### Organic Elements and Functional Groups in the Protodolomite

**FIGURE 11:**
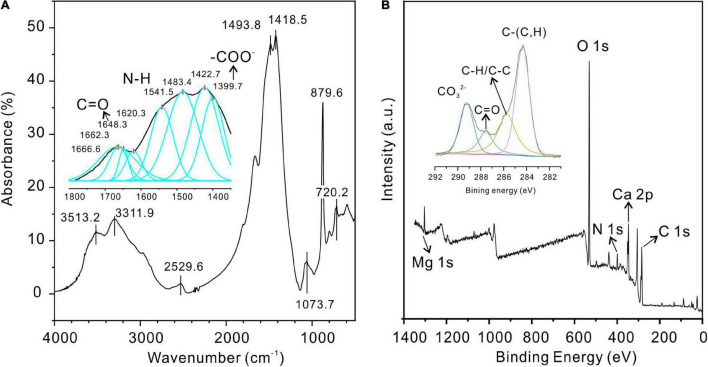
Fourier-transform infrared spectroscopy (FTIR) and XPS analyses of protodolomite induced by *Vibrio harveyi* QPL2. **(A)** FTIR; **(B)** XPS.

The precipitate induced by QPL2 in the medium with a Mg/Ca molar ratio of 12 was further identified as protodolomite by FTIR, XPS (Figure 11), and Raman spectroscopy (Supplementary Figure 4). The bi-dentated peak observed in the FTIR at 1,483 and 1,428 cm^–1^ indicates the presence of huntite in the precipitates, supporting the results of SEM (Figures 6A,B). The absence of a diffraction peak for huntite in the XRD pattern of the precipitates in this group confirms that the proportion of huntite is very small, which is unlikely to affect further analyses. The FTIR and XPS results of the protodolomite show visible organic signals (Figure 11). Three internal modes of CO_3_ groups were observed in the FTIR measurements, including ν_4_ (720.2 cm^–1^), v_2_ (879.6 cm^–1^), and v_3_ (1,418 cm^–1^). There are also several strong bands of water in the crystals including peaks located at 3,513.2, 3,311.9, and 2,529.6 cm^–1^, corresponding to the bound water detected by XRD (Figure 11A). In the enlarged inset figure, several organic bands can be recognized after the peak-fitting process, including peaks observed at 1,399.7, 1,541.5, and 1,648.3 cm^–1^, which are normally assigned to the –COO^–^, N–H, and C=O from amides of protein (Omoike and Chorover, 2004). The –OH group located at about 1,073.7 cm^–1^ from polysaccharide can also be recognized from the main pattern (Omoike and Chorover, 2004). The peaks of –COO^–^, N–H, and C=O proteins are also present in the infrared spectra of EPS, indicating that the organic functional groups of minerals may be closely related to EPS (Supplementary Figure 5A). The XPS results also show clear inorganic and organic ion signals (Figure 11B). Specifically, the Ca 2p core-level spectrum has two peaks, including Ca 2p 1/2 (350.41 eV) and Ca 2p 3/2 (346.71 eV) (Yuan et al., 2011). The Mg 1s core-level spectrum located at 1,303.71 eV corresponds to the Mg–O (Chen et al., 2012). The O 1s spectrum located at 531.11 eV is consistent with O–C=O (Supplementary Figures 5B–D; Han et al., 2019). The C 1s peaks observed at 284.31, 285.81, 287.51, and 289.21 eV are indicative of C–(C,H), C–H/C–C, C=O, and ${\text{CO}}_{3}^{2-}$, respectively (Figure 11B).

Further analysis of the organic molecules involved mineralization, deconvolution of high-resolution scans of P 2p and N 1s, and recognition of the corresponding functional groups. The P 2p peak located at 132.71 eV can be attributed to P-O. The N 1s peak is related to C-NH_2_ groups (399.41 eV) from organics (Supplementary Figures 5E,F; Rouxhet and Genet, 2011). These results further confirm that there is significant water and organics within the protodolomite particles induced by *Vibrio harveyi* QPL2, suggesting that organics were crucial in the growth of protodolomite.

### Role of Cells in the Precipitation of Protodolomite

#### Amino Acids and Polysaccharide From Extracellular Polymeric Substances of *Vibrio harveyi* QPL2

**FIGURE 12:**
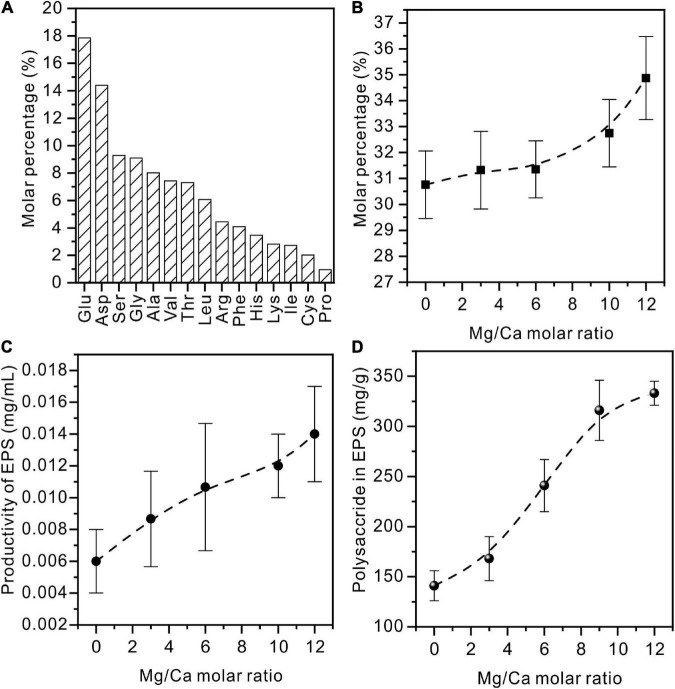
Amino acids and polysaccharides in EPS extracted from *Vibrio harveyi* QPL cultured in a medium with various Mg/Ca molar ratios. **(A)** Amino acids in EPS of QPL2 cultured in a medium with no addition of Ca^2+^ and Mg^2+^. **(B)** Acidic amino acids (Glu and Asp) in EPS with increasing Mg/Ca ratios. **(C)** The total amount of EPS with increasing Mg/Ca ratios. **(D)** The polysaccharide in EPS with increasing Mg/Ca ratios.

The freeze-dried pure EPS powder viewed under SEM is a uniform thin film (Supplementary Figure 6). The organic composition of the extracted EPS was further determined. From Figure 12A, it can be seen that acidic amino acids (Asp and Glu) form the highest proportion of all 15 kinds of amino acid. This is in line with the basic characteristic of halophilic bacteria (Fukuchi et al., 2003). The detailed data on amino acids in EPS extracted from medium with Mg/Ca molar ratios of 0, 3, 6, 10, and 12 are shown in Supplementary Figure 7. It was also found that these acidic amino acids increased with the increase in Mg^2+^ ions and reached the highest value at Mg/Ca = 12 (Figure 12B). The weight of EPS secreted by bacteria is related to the Mg^2+^ ions. With the increase of Mg^2+^ ion content, the quality of EPS gradually increases and more EPS is conducive to the nucleation of dolomite (Figure 12C).

Similar to the change in amino acids, the content of polysaccharides also increases with rising Mg^2+^ content (Figure 12D). Polysaccharide is a macromolecular substance connected by monosaccharide through a glycosidic bond. It is an important component of EPS with a complex structure. As the main component of acidic polysaccharides, uronic acid can act as the ligand of metal cations. After the EPS was derivatized and subjected to acidolysis, the monosaccharide composition is mainly neutral sugar. Acid sugar and partial neutral sugar increased significantly with the increase of Mg^2+^ content. With Mg/Ca = 12, high contents of glucuronic acid and galacturonic acid were recorded (Supplementary Figure 7). This suggests that the formation of dolomite may be also related to the content of acidic polysaccharides, especially uronic acid.

#### Ultra-Thin Sections of *Vibrio harveyi* QPL2 Cell

**FIGURE 13:**
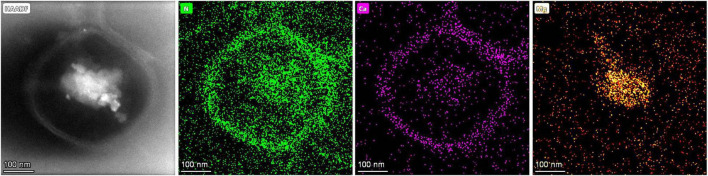
The typical elemental distribution of N, Ca, and Mg on an ultra-thin section of *Vibrio harveyi* QPL2 cell cultured under the condition of a Mg/Ca ratio of 12.

Figure 13 shows ultra-thin sections of *Vibrio harveyi* QPL2, cultured under the conditions of Mg/Ca ratio = 12. The element N is evenly distributed on the cell surface, showing the outline of the bacteria, whereas Ca^2+^ ions are mainly distributed within the cell wall and inside the cells. Magnesium ions are also diffused on the cell surface and are significantly enriched in the nucleus area of the cell, since Mg^2+^ acts as a counter ion for the nuclear acids (Jahnen-Dechent and Ketteler, 2012). These results clearly show that Ca^2+^ and Mg^2+^ ions are adsorbed onto the cell wall and within the EPS. This local concentration of ions may be one of the microenvironments required for the nucleation of primary dolomite.

## Discussion

In this study, protodolomite was precipitated in the presence of the aerobically cultured extreme halophilic bacterium *Vibrio harveyi* QPL2 in the medium with high Mg/Ca molar ratios of 10 and 12. However, no protodolomite was formed in the culture medium with relatively lower Mg/Ca ratios (0, 3, and 6). Considering that all of the experiments were conducted under the same conditions with the same bacterial strain, but not all precipitated protodolomite, the results clearly indicate that the high Mg/Ca ratio of the ambient solution is the key factor for promoting protodolomite precipitation in these experiments. Although several species of halophilic bacteria have been shown to have the ability to induce protodolomite or dolomite (Qiu et al., 2017), the different experimental designs in previous studies and the detailed results in this present research demonstrate several new factors in the precipitation of bacterially-induced protodolomite.

### Cation Ordering and Stoichiometry of Protodolomite Induced by *Vibrio harveyi* QPL2

Different from calcite and its isomorphisms (Mg-calcite with variable Mg content including low Mg-calcite, high Mg-calcite, and very high Mg-calcite), the arrangement of atoms in the dolomite crystal structure is unique (Warren, 2000; Zhang et al., 2021). In dolomite crystals, separate layers of Ca^2+^ and Mg^2+^ cations alternate with ${\text{CO}}_{3}^{2-}$ anion layers; this arrangement gives rise to the particular ordering or super-lattice structure of the crystals (Warren, 2000; Gregg et al., 2015; Qiu et al., 2017). Thus, there is no center in the symmetry of dolomite (${\text{R}}_{3}^{-}$) compared to that of calcite or Mg-calcite ($R_{3}^{-}\,c$) (Gregg et al., 2015). The ordering peaks are the indispensable characteristic of dolomite, used to distinguish it from very high magnesium calcite (VHMC). Based on this fact, some microbially induced precipitates in previous studies were termed protodolomite (VHMC) rather than ordered dolomite (Gregg et al., 2015). In the present study, only a simulated ordering reflection (015) that is obscured by background noise is identified, whereas (101) and (021) are absent (Figures 4, 5). Thus, on the whole, the precipitate induced by *Vibrio harveyi* QPL2 is not completely ordered dolomite.

There may be several reasons for the difficulty of the “ordering” structure present. Qiu et al. (2017) believed that a relatively short reaction time may be the primary reason for the disorder of microbial-mediated dolomite, since both modeling work (Arvidson and Mackenzie, 1999) and the nature of ancient dolomite show a relationship between ordering structure and formation time (Gregg et al., 1992). In the present case, we suggest the rapid growth speed is another key factor for the presence of the disordered structure. This is similar to the formation process of “amorphous solids” in classic crystallography, which can simply be interpreted as the atoms being fixed before they reached the space point with the lowest energy in the crystal. Several experiment results here point to rapid growth of the protodolomite obtained, including weak crystallinity (Figure 4), micron-sized protodolomite spheres (Figure 6), loose surface structure (Figures 6, 8), and high water and organic contents (Figures 10, 11) in the crystal. Although the rapid growth of crystals is not conducive to the appearance of an ordered structure, it is beneficial for more Mg^2+^ ions to replace Ca^2+^ ions in the crystal structure in the form of isomorphism.

**FIGURE 14:**
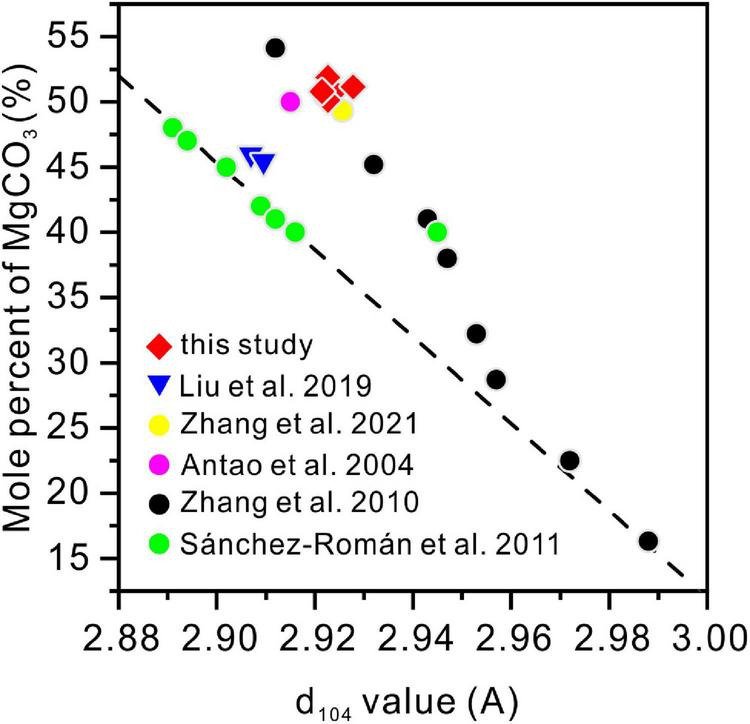
The relationship between the d_104_ values and the MgCO_3_ volume in the magnesium calcite isomorphism series from this and previous studies. The straight dashed line is the linear function (N_MgCO_3__ = B-M × d_104_, where N_MgCO_3__ is the molar percentage of MgCO_3_ in the isomorphisms, d104 is the observed (104) interplanar spacing (Å), B = 1011.99, M = 333.33).

The stoichiometry of the protodolomite refers to the MgCO_3_ content in the crystal. The ideal stoichiometry of dolomite is 50%. In previous research, the content of MgCO_3_ in bacterially-induced dolomite (protodolomite) is usually lower than that of CaCO_3_ (Sánchez-Román et al., 2011; Qiu et al., 2017). However, in the present results, we found that the MgCO_3_ in the protodolomite induced by *Vibrio harveyi* QPL2 is slightly more enriched than the CaCO_3_ (Figure 8). It is likely that there are at least two reasons for this difference. First, the method (function) adopted to calculate the Mg^2+^ ion content was different. Most of the previous studies used the linear relationship between increasing Mg^2+^ content and a decrease in the (104) d-spacing (larger 2θ value) to perform the approximate calculations (the dash line in Figure 14). N_MgCO_3__ = B-M × d_104_ (1), where N_MgCO_3__ is molar percentage of MgCO_3_ in the isomorphism, d_104_ is the observed (104) interplanar spacing (Å), B = 1011.99, M = 333.33). This function was first deduced by Chave (1952) in skeletal calcites containing up to 30 mol% excess MgCO_3_. There are other variations of this formula (Goldsmith and Graf, 1958; Runnells, 1970). However, later experiments have shown that when the content of magnesium in Mg-calcite is more than 30%, the formula will produce deviation (Zhang et al., 2010). Our data locate in the empirical curve proposed by Zhang et al. (2010) between d_104_ values and composition in the Mg-calcite isomorphism series (Figure 14). The second possible reason may be that the Mg/Ca ratios used in the present protodolomite precipitation experiments are higher (10 and 12), and these may have enhanced the ability of Mg^2+^ ions to compete with Ca^2+^ ions (Aoba et al., 1992), thereby allowing more Mg^2+^ ions to enter the mineral structure. In general, the protodolomite induced by extreme halophilic bacteria QPL2 in the present study is a disordered very high magnesium calcite (i.e., VHMC) with a slight excess of Mg^2+^ compared to ideal dolomite.

### High Mg/Ca Molar Ratios Promote the Precipitation of Halophilic Bacterially Induced Protodolomite

It is widely accepted that EPS protect bacteria by gel formation, ion exchange, mineral formation, and the accumulation of toxic metal ions (Flemming and Wingender, 2010). In the current experiments, the increase in the ratio of Mg/Ca in the solution triggered the self-protection mechanism of bacteria, leading to the production of more EPS (Figure 12C). The extremely high salt concentration in the living environment forces the EPS composition on the surface of halophilic bacteria to be clearly distinguished from the microorganisms living in the normal environment (Fukuchi et al., 2003). Numerous studies have suggested that the acidic amino acids are richer in halophilic bacteria compared to those of non-halophilic bacteria (Fukuchi et al., 2003; Qiu et al., 2017), because the high content of acidic amino acids (such as aspartic acid and glutamic acid) can form a negatively-charged protective layer on the cell surface to attract positively-charged ions, thereby maintaining cell-wall stability and preventing cell lysis (Lanyi, 1974; Rao and Argos, 1981). The present experiments confirm that acidic amino acids (Glu and Asp) make up nearly one-third of all amino acids (Figure 12A). As the Mg/Ca ratio increases, the acidic amino acid and polysaccharide contents in EPS also increase, and this may be the key factor in protodolomite precipitation. This relationship is similar to that between salinity (NaCl) and carboxyl reported by Qiu et al. (2017). Therefore, the most likely reason for the absence of protodolomite in the medium with a Mg/Ca molar ratio of 3 and 6 may be that the density of the organic functional groups does not reach a threshold for precipitation.

In solution, both Ca^2+^ and Mg^2+^ ions are hydrated with H_2_O to form hydrous complexes, so they need to undergo a dehydration process before they can enter the anhydrous mineral structure. The dehydration process of Mg^2+^ is more difficult than that of Ca^2+^. There are at least two reasons: (1) the enthalpy of Mg^2+^ dehydration (ΔH = 351.8 kcal/mol for $\text{Mg}\,{\left[{\text{H}}_{2}\,\text{O}\right]}_{6}^{2}$ at 298 K, 1 atm) is significantly higher than that of Ca^2+^ dehydration (ΔH = 264.3 kcal/mol for $\text{Ca}\,{\left[{\text{H}}_{2}\,\text{O}\right]}_{6}^{2}$ at 298 K, 1 atm) (Katz et al., 1996); (2) The half-life of Mg-H_2_O (600 ps) is significantly longer than that of Ca-H_2_O (19 ps) (Jiao et al., 2006). The mechanism by which acidic amino acids can promote the precipitation of protodolomite is that their rich carboxyl groups can replace the water molecules in Mg-H_2_O complexes (Roberts et al., 2013), and this requires a lower energy for carbonation and the subsequent growth of ions (Romanek et al., 2009; Roberts et al., 2013). Polysaccharides can also accelerate Mg dehydration and subsequent crystal nucleation because of their high content of hydroxyl groups (Liu et al., 2020). In this way, a large amount of Ca^2+^ and Mg^2+^ ions accumulate within the EPS (Figure 13) and are combined with ${\text{CO}}_{3}^{2-}$ generated by the bacterial activity to form Ca-Mg carbonate nuclei.

**FIGURE 15:**
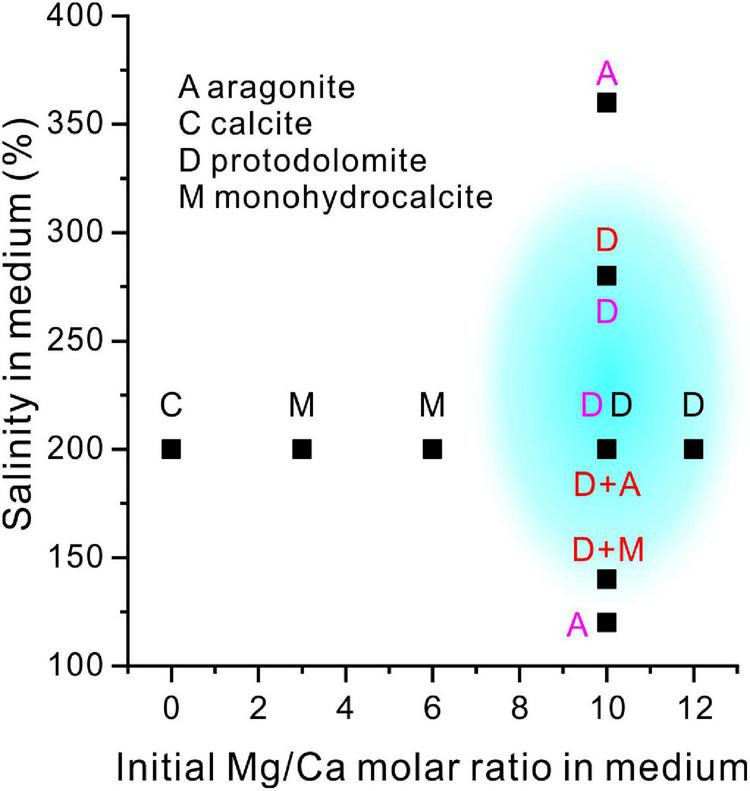
A comparison of the present experiment results with previous studies of mineralogy with salinity and increasing Mg/Ca ratios. The blue shadow shows the possible formation conditions of the extreme halophilic bacterially-induced protodolomite. Letters in a black, red and purple color are the results of the present study, the results of Qiu et al. (2017) and the results of Qiu et al. (2019), respectively.

By comparing the results of our experiments with those of the extreme halophilic bacteria *Haloferax volcanii* DS52 (Qiu et al., 2017) and *Natrinema* sp. J7-1 (Qiu et al., 2019; Figure 15), it clearly shows that the precipitation of halophilic bacterially induced protodolomite is promoted by high salinity and Mg/Ca molar ratios (blue area in Figure 15).

Taken together, our results suggest that high Mg/Ca molar ratios not only constrain the saturation of dolomite in the solution but also change the organic components of the EPS which are closely related to the protodolomite precipitation. The present results show that an increase in Mg/Ca molar ratio will lead to an increase in acidic amino acids and polysaccharides in the EPS; these are the facilitators of protodolomite precipitation.

### Proposed Model of Halophilic Bacterially-Induced Protodolomite Precipitation

Based on our experiments, the route of halophilic bacterially induced protodolomite precipitation can be summarized. In the initial solution, the pH of the medium is about 7.0, and no carbonate minerals are precipitated under this condition (Figure 3C). The alkaline nature of the medium begins to develop when the QPL2 grows rapidly to produce ammonia and carbonic anhydrase and this has been confirmed in several of our previous studies (Zhuang et al., 2018; Zhao et al., 2019, 2021). With a medium with a high Mg/Ca ratio of 10 and 12, protodolomite is then able to precipitate quickly after crystal nuclei formation on the cell surface. Thus, some bacterial cells become enveloped in a mineral crust (Figure 6L), whereas others are enclosed within the mineral spheres.

Protodolomite induced by QPL2 is always in the form of spheres. All things considered, the spherical growth mode has a lower Gibbs free energy, which is conducive to the rapid increase in mineral volume (Joshi and Antony, 1979; Zhang et al., 2017). Conversely, this is also the result of the adsorption of various organic molecules on the mineral crystal surface. The adsorption on different crystal planes may reduce the difference in crystal growth rate, which is not conducive to the appearance of crystal planes (Meldrum and Hyde, 2001; Zhao et al., 2021).

### Geological Implications

In the geological record, most of the sedimentary dolomites in carbonate rocks are ordered (Warren, 2000), whereas most of the dolomite (protodolomite) minerals produced by the action of microorganisms in these and previous studies are usually disordered (Sánchez-Román et al., 2009; Gregg et al., 2015; Qiu et al., 2017; Liu et al., 2020; Zhang et al., 2021). Even if the original dolomite can exist for a long time in an organic-rich environment (Meldrum and Hyde, 2001), based on the basic principles of crystallography, the original dolomite produced in all these experiments is obviously a thermodynamically metastable mineral (Warren, 2000). There are at least two transformation directions to reach the stable phases. The first is the gradual leakage of Mg^2+^ from the protodolomite, such that it develops into a stable calcite or low-Mg calcite (<5 mol% MgCO_3_). The other is the self-regulation of Mg^2+^, whereby order is gradually established to form the ideal dolomite. Burial processes, with increasing temperature and pressure, may have a positive effect on the transformation of protodolomite into ordered dolomite. The high bound-water content in protodolomite (as in Figure 10) can catalyze the ordering process to dolomite under higher temperature (Zheng et al., 2021). At the same time, data from the geological record show that burial depth (and time) is positively correlated with the increased ordering and stoichiometry of dolomite (Gregg et al., 1992; Warren, 2000). Thus, it is most likely that some protodolomite induced by microorganisms is transformed into “classic” dolomite through recrystallization.

The organic-rich nature of initial dolomite precipitates may have an organic geochemical significance. The results of the experiment reported here show that the percentage of organic matter in the newly-formed protodolomite is about 5 wt% (Figure 10). Since most of this organic matter is physically enclosed within minerals, rapid oxidation may be reduced, allowing its preservation. Consequently, the organic matter may be converted into kerogen or hydrocarbons under increasing temperature and pressure in a deeper burial environment. Numerous studies have shown that many primary microbial dolostones are important hydrocarbon sources and reservoir rocks (Słowakiewicz et al., 2013; Zhu et al., 2020).

## Conclusion

A newly isolated extreme halophilic strain *Vibrio harveyi* QPL2 from a modern saline environment was utilized in biomineralization experiments in a medium with 200‰ and various Mg/Ca molar ratios. It was found that protodolomite could be induced in the mediums with the Ma/Ca molar ratios of 10 and 12. The spherical protodolomite precipitated with pinhole and micro-globule structures is stoichiometric but with no obvious crystal lattice ordering. Bound water and a high organic content occur within the protodolomite. Both the hydrochemical conditions and bacterial activities facilitate the formation of protodolomite, as a result of the high Mg/Ca molar ratios increasing the amounts of acidic amino acids and polysaccharides in the EPS, both of which are facilitators of protodolomite precipitation.

## Data Availability Statement

The datasets presented in this study can be found in online repositories. The names of the repository/repositories and accession number(s) can be found below: https://www.ncbi.nlm.nih.gov/genbank/, OK493384.

## Author Contributions

ZH: conceptualization, data curation, funding acquisition, and methodology. PQ: formal analysis and investigation. YZ: writing—original draft, investigation, methodology, and funding acquisition. NG: investigation and methodology. HY: data curation and methodology. MT: English quality. DL and HZ: investigation. All authors contributed to the article and approved the submitted version.

## Conflict of Interest

The authors declare that the research was conducted in the absence of any commercial or financial relationships that could be construed as a potential conflict of interest.

## Publisher’s Note

All claims expressed in this article are solely those of the authors and do not necessarily represent those of their affiliated organizations, or those of the publisher, the editors and the reviewers. Any product that may be evaluated in this article, or claim that may be made by its manufacturer, is not guaranteed or endorsed by the publisher.

## References

[B1] Al Disi Z. A. Bontognali T. R. R. Jaoua S. Attia E. Al-Kuwari H. A. S. Zouari N. (2019). Influence of temperature, salinity and Mg2+: Ca2+ ratio on microbially-mediated formation of Mg-rich carbonates by *Virgibacillus* strains isolated from a sabkha environment. *Sci. Rep.* 9 1–12. 10.1038/s41598-019-56144-0 31873136 PMC6928219

[B2] Al Disi Z. A. Jaoua S. Bontognali T. R. R. Attia E. S. M. Al-Kuwari H. A. A. S. Zouari N. (2017). Evidence of a role for aerobic bacteria in high magnesium carbonate formation in the evaporitic environment of Dohat Faishakh Sabkha in Qatar. *Front. Environ. Sci.* 5:1. 10.3389/fenvs.2017.00001

[B3] Aoba T. Moreno E. C. Shimoda S. (1992). Competitive adsorption of magnesium and calcium ions onto synthetic and biological apatites. *Calcif. Tissue Int* 51 143–150. 10.1007/BF00298503 1422954

[B4] Arvidson R. S. Mackenzie F. T. (1999). The dolomite problem; control of precipitation kinetics by temperature and saturation state. *Am. J. Sci.* 299 257–288. 10.2475/ajs.299.4.257 12377050

[B5] Baker P. A. Kastner M. (1981). Constraints on the formation of sedimentary dolomite. *Science* 213 214–216. 10.1126/science.213.4504.214 17782787

[B6] Baldermann A. Deditius A. P. Dietzel M. Fichtner V. Fischer C. Hippler D. (2015). The role of bacterial sulfate reduction during dolomite precipitation: implications from Upper Jurassic platform carbonates. *Chem. Geol.* 412 1–14. 10.1016/j.chemgeo.2015.07.020

[B7] Baxter B. K. (2018). Great Salt Lake microbiology: a historical perspective. *Int. Microbiol.* 21 79–95. 10.1007/s10123-018-0008-z 30810951 PMC6133049

[B8] Bazaka K. Crawford R. J. Nazarenko E. L. Ivanova E. P. (2011). Bacterial extracellular polysaccharides. *Adv. Exp. Med. Biol.* 715:213. 10.1007/978-94-007-0940-9_13 21557066

[B9] Bontognali T. R. R. McKenzie J. A. Warthmann R. J. Vasconcelos C. (2014). Microbially influenced formation of Mg-calcite and Ca-dolomite in the presence of exopolymeric substances produced by sulphate-reducing bacteria. *Terra. Nova* 26 72–77. 10.1111/ter.12072

[B10] Bontognali T. R. R. Vasconcelos C. Warthmann R. J. Bernasconi S. M. Dupraz C. Strohmenger C. J. (2010). Dolomite formation within microbial mats in the coastal sabkha of Abu Dhabi (United Arab Emirates). *Sedimentology* 57 824–844. 10.1111/j.1365-3091.2009.01121.x

[B11] Bontognali T. R. Vasconcelos C. Warthmann R. J. Lundberg R. McKenzie J. A. (2012). Dolomite-mediating bacterium isolated from the sabkha of Abu Dhabi (UAE). *Terra Nova* 24 248–254. 10.1111/j.1365-3121.2012.01065.x

[B12] Borsodi A. K. Felföldi T. Máthé I. Bognár V. Knáb M. Krett G. (2013). Phylogenetic diversity of bacterial and archaeal communities inhabiting the saline/Lake Red located in Sovata, Romania. *Extremophiles* 17 87–98. 10.1007/s00792-012-0496-2 23132551

[B13] Braithwaite C. J. Rizzi G. Darke G. (2004). The geometry and petrogenesis of dolomite hydrocarbon reservoirs: introduction. *Geol. Soc. Lond. Special Publ.* 235 1–6. 10.1144/GSL.SP.2004.235.01.01

[B14] Brantley S. L. (2003). Reaction kinetics of primary rock-forming minerals under ambient conditions. *Treatise Geochem.* 5:605. 10.1016/b978-0-08-095975-7.00503-9

[B15] Burns S. J. Mckenzie J. A. Vasconcelos C. (2000). Dolomite formation and biogeochemical cycles in the Phanerozoic. *Sedimentology* 47 49–61. 10.1046/j.1365-3091.2000.00004.x

[B16] Chave K. E. (1952). A solid solution between calcite and dolomite. *J. Geol.* 60 190–192. 10.2307/30061254

[B17] Chen J. Song Y. Shan D. Han E. H. (2012). Study of the in situ growth mechanism of mg–al hydrotalcite conversion film on az31 magnesium alloy. *Corrosion Sci.* 63 148–158. 10.1016/j.corsci.2012.05.022

[B18] de Leeuw N. H. Parker S. C. (2001). Surface–water interactions in the dolomite problem. *Phys. Chem. Chem. Phys.* 3 3217–3221. 10.1039/B102928M

[B19] de Lourdes Moreno M. Sánchez-Porro C. García M. T. Mellado E. (2012). Carotenoids’ production from halophilic bacteria. *Methods Mol. Biol.* 892:207. 10.1007/978-1-61779-879-5_12 22623305

[B20] Deckker P. D. Last W. M. (1988). Modern dolomite deposition in continental, saline lakes, western Victoria, Australia. *Geology* 16 29–32.

[B21] Deng S. Dong H. Lv G. Jiang H. Yu B. Bishop M. E. (2010). Microbial dolomite precipitation using sulfate reducing and halophilic bacteria: results from Qinghai Lake, Tibetan Plateau, NW China. *Chem. Geol.* 278 151–159. 10.1016/j.chemgeo.2010.09.008

[B22] Diloreto Z. A. Garg S. Bontognali T. R. R. Dittrich M. (2021). Modern dolomite formation caused by seasonal cycling of oxygenic phototrophs and anoxygenic phototrophs in a hypersaline sabkha. *Sci. Rep. U.K.* 11:4170. 10.1038/s41598-021-83676-1 33603064 PMC7893050

[B23] Flemming H.-C. Wingender J. (2010). The biofilm matrix. *Nat. Rev. Microbiol.* 8 623–633. 10.1038/nrmicro2415 20676145

[B24] Foti M. Sorokin D. Y. Lomans B. Mussman M. Zacharova E. E. Pimenov N. V. (2007). Diversity, activity, and abundance of sulfate-reducing bacteria in saline and hypersaline soda lakes. *Appl. Environ. Microb.* 73 2093–2100. 10.1128/AEM.02622-06 17308191 PMC1855663

[B25] Fukuchi S. Yoshimune K. Wakayama M. Moriguchi M. Nishikawa K. (2003). Unique amino acid composition of proteins in halophilic bacteria. *J. Mol. Biol.* 327 347–357. 10.1016/S0022-2836(03)00150-5 12628242

[B26] Goldsmith J. R. Graf D. L. (1958). Structural and compositional variations in some natural dolomites. *J. Geol.* 66 678–693. 10.1086/626547

[B27] Gregg J. M. Bish D. L. Kaczmarek S. E. Machel H. G. (2015). Mineralogy, nucleation and growth of dolomite in the laboratory and sedimentary environment: a review. *Sedimentology* 62 1749–1769. 10.1111/sed.12202

[B28] Gregg J. M. Howard S. A. Mazzullo S. J. (1992). Early diagenetic recrystallization of Holocene (<3000 years old) peritidal dolomites, Ambergris Cay, Belize. *Sedimentology* 39 143–160. 10.1111/j.1365-3091.1992.tb01027.x

[B29] Han L. Yao Q. Z. Wang F. P. Huang Y. R. Fu S. Q. Zhou G. T. (2019). Insights into the formation mechanism of vaterite mediated by a deep-sea bacterium *shewanella piezotolerans* wp3. *Geochim. Cosmochim. Acta* 256 35–48. 10.1016/j.gca.2018.06.011

[B30] Hatakeyama T. Nakamura K. Hatakeyama H. (1988). Determination of bound water content in polymers by DTA, DSC and TG. *Thermochim. Acta* 123 153–161. 10.1016/0040-6031(88)80018-2

[B31] Jahnen-Dechent W. Ketteler M. (2012). Magnesium basics. *Clin. Kidney J.* 5 (Suppl 1) i3–i14. 10.1093/ndtplus/sfr163 26069819 PMC4455825

[B32] Jiang H. Dong H. Yu B. Liu X. Li Y. Ji S. (2007). Microbial response to salinity change in Lake Chaka, a hypersaline lake on Tibetan plateau. *Environ. Microbiol.* 9 2603–2621. 10.1111/j.1462-2920.2007.01377.x 17803783

[B33] Jiang H. Dong H. Zhang G. Yu B. Chapman L. R. Fields M. W. (2006). Microbial diversity in water and sediment of lake chaka, an athalassohaline lake in Northwestern China. *Appl. Environ. Microb.* 72 3832–3845. 10.1128/AEM.02869-05 16751487 PMC1489620

[B34] Jiao D. King C. Grossfield A. Darden T. A. Ren P. (2006). Simulation of Ca2+ and Mg2+ solvation using polarizable atomic multipole potential. *J. Phys. Chem. B* 110 18553–18559. 10.1021/jp062230r 16970483

[B35] Joshi M. S. Antony A. V. (1979). Nucleation in supersaturated potassium dihydrogen orthophosphate solutions. *J. Cryst. Growth* 46 7–9. 10.1016/0022-0248(79)90100-3

[B36] Katz A. K. Glusker J. P. Beebe S. A. Bock C. W. (1996). Calcium ion coordination: a comparison with that of beryllium, magnesium, and zinc. *J. Am. Chem. Soc.* 118 5752–5763. 10.1021/ja953943i

[B37] Kohut C. Dudas M. Muehlenbachs K. (1995). Authigenic dolomite in a saline soil in Alberta, Canada. *Soil Sci. Soc. Am. J.* 59 1499–1504.

[B38] Kolpakova M. N. Gaskova O. L. Naymushina O. S. Karpov A. V. Vladimirov A. G. Krivonogov S. K. (2019). Saline lakes of Northern Kazakhstan: geochemical correlations of elements and controls on their accumulation in water and bottom sediments. *Appl. Geochem.* 107 8–18. 10.1016/j.apgeochem.2019.05.013

[B39] Land L. S. (1998). Failure to precipitate dolomite at 25 °C from dilute solution despite 1000-fold oversaturation after 32 Years. *Aquat. Geochem.* 4 361–368. 10.1023/A:1009688315854

[B40] Lanyi J. K. (1974). Salt-dependent properties of proteins from extremely halophilic bacteria. *Bacteriol. Rev.* 38 272–290. 10.1128/BR.38.3.272-290.1974 4607500 PMC413857

[B41] Li J. Zhu L. Li M. Wang J. Ma Q. (2020). Origin of modern dolomite in surface lake sediments on the central and western Tibetan Plateau. *Quat. Int.* 544 65–75. 10.1016/j.quaint.2020.02.018

[B42] Lippmann F. (1982). Stable and metastable solubility diagrams for the system CaCO3-MgCO3-H2O at ordinary temperature. *Bull. Mineral.* 105 273–279.

[B43] Liu D. Fan Q. Papineau D. Yu N. Chu Y. Wang H. (2020). Precipitation of protodolomite facilitated by sulfate-reducing bacteria: the role of capsule extracellular polymeric substances. *Chem. Geol.* 533:119415. 10.1016/j.chemgeo.2019.119415

[B44] Liu D. Yu N. Papineau D. Fan Q. Wang H. Qiu X. (2019). The catalytic role of planktonic aerobic heterotrophic bacteria in protodolomite formation: results from Lake Jibuhulangtu Nuur, Inner Mongolia, China. *Geochim. Cosmochim. Acta* 263 31–49. 10.1016/j.gca.2019.07.056

[B45] Malone M. J. Baker P. A. Burns S. J. (1996). Recrystallization of dolomite: an experimental study from. *Geochim. Cosmochim. Acta* 60 2189–2207. 10.1016/0016-7037(96)00062-2

[B46] McKenzie J. A. Vasconcelos C. (2009). Dolomite Mountains and the origin of the dolomite rock of which they mainly consist: historical developments and new perspectives. *Sedimentology* 56 205–219. 10.1111/j.1365-3091.2008.01027.x

[B47] Meldrum F. C. Hyde S. T. (2001). Morphological influence of magnesium and organic additives on the precipitation of calcite. *J. Cryst. Growth* 231 544–558. 10.1016/S0022-0248(01)01519-6

[B48] Morgan J. W. Forster C. F. Evison L. (1990). A comparative study of the nature of biopolymers extracted from anaerobic and activated sludges. *Water Res.* 24 743–750. 10.1016/0043-1354(90)90030-A

[B49] Omoike A. Chorover J. (2004). Spectroscopic study of extracellular polymeric substances from bacillus subtilis: aqueous chemistry and adsorption effects. *Biomacromolecules* 5 1219–1230. 10.1021/bm034461z 15244434

[B50] Oren A. (2008). Microbial life at high salt concentrations: phylogenetic and metabolic diversity. *Saline Syst.* 4:2. 10.1186/1746-1448-4-2 18412960 PMC2329653

[B51] Pan J. Zhao H. Tucker M. E. Zhou J. Jiang M. Wang Y. (2019). Biomineralization of monohydrocalcite induced by the halophile *Halomonas smyrnensis* WMS-3. *Minerals* 9:632. 10.3390/min9100632

[B52] Perri E. Tucker M. (2007). Bacterial fossils and microbial dolomite in *Triassic stromatolites*. *Geology* 35 207–210. 10.1130/g23354a.1

[B53] Qiu X. Wang H. Yao Y. Duan Y. (2017). High salinity facilitates dolomite precipitation mediated by *Haloferax volcanii* DS52. *Earth Planet. Sci. Lett.* 472 197–205. 10.1016/j.epsl.2017.05.018

[B54] Qiu X. Yao Y. Wang H. Shen A. Zhang J. (2019). Halophilic archaea mediate the formation of proto-dolomite in solutions with various sulfate concentrations and salinities. *Front. Microbiol.* 10:480. 10.3389/fmicb.2019.00480 30915060 PMC6422947

[B55] Rao J. M. Argos P. (1981). Structural stability of halophilic proteins. *Biochemistry* 20 6536–6543. 10.1021/bi00526a004 6796115

[B56] Roberts J. A. Kenward P. A. Fowle D. A. Goldstein R. H. González L. A. Moore D. S. (2013). Surface chemistry allows for abiotic precipitation of dolomite at low temperature. *Proc. Natl. Acad. Sci. U.S.A.* 110 14540–14545. 10.1073/pnas.1305403110 23964124 PMC3767548

[B57] Rodriguez-Blanco J. D. Shaw S. Benning L. G. (2015). A route for the direct crystallization of dolomite. *Am. Mineral.* 100 1172–1181. 10.2138/am-2015-4963

[B58] Romanek C. S. Jiménez-López C. Navarro A. R. Sánchez-Román M. Sahai N. Coleman M. (2009). Inorganic synthesis of Fe–Ca–Mg carbonates at low temperature. *Geochim. Cosmochim. Acta* 73 5361–5376. 10.1016/j.gca.2009.05.065

[B59] Rouxhet P. G. Genet M. J. (2011). XPS analysis of bio-organic systems. *Surface Interface Anal.* 43 1453–1470. 10.1002/sia.3831

[B60] Runnells D. D. (1970). Errors in x-ray analysis of carbonates due to solid-solution variation in composition of component minerals. *J. Sediment. Res.* 40 1158–1166.

[B61] Samylina O. S. Zaytseva L. V. (2019). Characterization of modern dolomite stromatolites from hypersaline Petukhovskoe Soda Lake, Russia. *Lethaia* 52 1–13. 10.1111/let.12286

[B62] Sánchez-Román M. McKenzie J. A. de Luca Rebello Wagener A. Rivadeneyra M. A. Vasconcelos C. (2009). Presence of sulfate does not inhibit low-temperature dolomite precipitation. *Earth Planet. Sci. Lett.* 285 131–139. 10.1016/j.epsl.2009.06.003

[B63] Sánchez-Román M. Romanek C. S. Fernández-Remolar D. C. Sánchez-Navas A. McKenzie J. A. Pibernat R. A. (2011). Aerobic biomineralization of Mg-rich carbonates: Implications for natural environments. *Chem. Geol.* 281 143–150. 10.1016/j.chemgeo.2010.11.020

[B64] Shalev N. Bontognali T. R. R. Vance D. (2020). Sabkha dolomite as an archive for the magnesium isotope composition of seawater. *Geology* 49 253–257. 10.1130/g47973.1

[B65] Sherman G. D. Kanehiro Y. Fujimoto C. K. (1947). *Dolomitization In Semi-Arid Hawaiian soils.* Honolulu, HI: University of Hawaii Press.

[B66] Słowakiewicz M. Tucker M. E. Pancost R. D. Perri E. Mawson M. (2013). Upper Permian (Zechstein) microbialites: supratidal through deep subtidal deposition, source rock, and reservoir potential. *AAPG Bull.* 97 1921–1936. 10.1306/06181312179

[B67] Sun F. Hu W. Wang X. Cao J. Fu B. Wu H. (2020). Methanogen microfossils and methanogenesis in Permian lake deposits. *Geology* 49 13–18. 10.1130/g47857.1

[B68] Vasconcelos C. McKenzie J. A. (1997). Microbial mediation of modern dolomite precipitation and diagenesis under anoxic conditions (Lagoa Vermelha, Rio de Janeiro, Brazil). *J. Sediment. Res.* 67 378–390. 10.1306/D4268577-2B26-11D7-8648000102C1865D

[B69] Vasconcelos C. McKenzie J. A. Bernasconi S. Grujic D. Tiens A. J. (1995). Microbial mediation as a possible mechanism for natural dolomite formation at low temperatures. *Nature* 377 220–222. 10.1038/377220a0

[B70] Warren J. (2000). Dolomite: occurrence, evolution and economically important associations. *Earth Sci. Rev.* 52 1–81. 10.1016/S0012-8252(00)00022-2

[B71] You X. Sun S. Zhu J. Li Q. Hu W. Dong H. (2013). Microbially mediated dolomite in Cambrian stromatolites from the Tarim Basin, north-west China: implications for the role of organic substrate on dolomite precipitation. *Terra. Nova* 25 387–395. 10.1111/ter.12048

[B72] Yuan S.-J. Sun M. Sheng G.-P. Li Y. Li W.-W. Yao R.-S. (2011). Identification of key constituents and structure of the extracellular polymeric substances excreted by *Bacillus megaterium* TF10 for their flocculation capacity. *Environ. Sci. Technol.* 45 1152–1157. 10.1021/es1030905 21174469

[B73] Zhang C. Lv J. Li F. Li X. (2017). Nucleation and growth of Mg-calcite spherulites induced by the bacterium *Curvibacter lanceolatus* strain HJ-1. *Microsc. Microanal.* 23 1189–1196. 10.1017/S1431927617012715 29199632

[B74] Zhang F. Xu H. Konishi H. Roden E. E. (2010). A relationship between d104 value and composition in the calcite-disordered dolomite solid-solution series. *Am. Mineral.* 95 1650–1656. 10.2138/am.2010.3414

[B75] Zhang F. Xu H. Shelobolina E. S. Konishi H. Roden E. E. (2021). Precipitation of low-temperature disordered dolomite induced by extracellular polymeric substances of methanogenic Archaea *Methanosarcina barkeri*: Implications for sedimentary dolomite formation. *Am. Mineral.* 106 69–81. 10.2138/am-2020-7381

[B76] Zhang F. Xu H. Shelobolina E. S. Konishi H. Converse B. Shen Z. (2015). The catalytic effect of bound extracellular polymeric substances excreted by anaerobic microorganisms on Ca-Mg carbonate precipitation: implications for the “dolomite problem”. *Am. Mineral.* 100 483–494. 10.2138/am-2015-4999

[B77] Zhao Y. Han Z. Yan H. Zhao H. Tucker M. E. Gao X. (2021). Selective adsorption of amino acids in crystals of monohydrocalcite induced by the facultative anaerobic *Enterobacter ludwigii* SYB1. *Front. Microbiol.* 12:696557. 10.3389/fmicb.2021.696557 34394038 PMC8358455

[B78] Zhao Y. Yan H. Zhou J. Tucker M. E. Han M. Zhao H. (2019). Bio-precipitation of calcium and magnesium ions through extracellular and intracellular process induced by *Bacillus licheniformis* SRB2. *Minerals* 9:526. 10.3390/min9090526

[B79] Zheng W. Liu D. Yang S. Fan Q. Papineau D. Wang H. (2021). Transformation of protodolomite to dolomite proceeds under dry-heating conditions. *Earth Planet. Sci. Lett.* 576:117249. 10.1016/j.epsl.2021.117249

[B80] Zhu D. Liu Q. He Z. Ding Q. Wang J. (2020). Early development and late preservation of porosity linked to presence of hydrocarbons in Precambrian microbialite gas reservoirs within the Sichuan Basin, southern China. *Precambrian Res.* 342:105694. 10.1016/j.precamres.2020.105694

[B81] Zhuang D. Yan H. Tucker M. E. Zhao H. Han Z. Zhao Y. (2018). Calcite precipitation induced by *Bacillus cereus* MRR2 cultured at different Ca2+ concentrations: further insights into biotic and abiotic calcite. *Chem. Geol.* 500 64–87. 10.1016/j.chemgeo.2018.09.018

