# Supplementary material for: High Mg/Ca Molar Ratios Promote Protodolomite Precipitation Induced by the Extreme Halophilic Bacterium Vibrio harveyi QPL2
Source: Front Microbiol. 2022 Apr 5;13:821968. doi: 10.3389/fmicb.2022.821968 (PMC9016281; doi:10.3389/fmicb.2022.821968)
Supplement: Supplementary file 1 [file Data_Sheet_1.PDF]

Table 1 The components in the medium prepared for biomineralization experiments.

Batch	Initial pH	Final pH	Initial Ca^{2+} (mol/L)	Initial Mg^{2+} (mol/L)	Mg/Ca molar ratio	NaCl concentration (wt/vt ‰)	Cell suspension (mL)
1	6.84	8.12	0.01	0	0	200	5
2	6.83	8.24	0.01	0.03	3	200	5
3	6.81	8.21	0.01	0.06	6	200	5
4	6.83	8.19	0.01	0.1	10	200	5
5	6.82	8.21	0.01	0.12	12	200	5

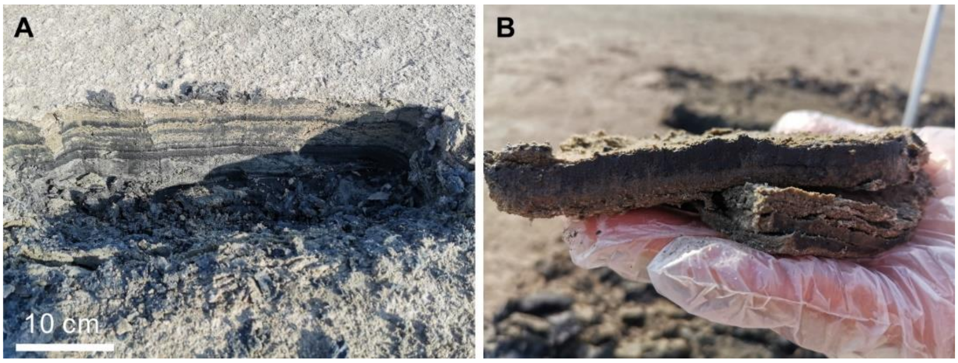

Figure 1 Images of the sample site at the Yinjiashan Salt Farm. (**A**) Section through the sediments; (**B**) The sample from which the halophilic bacteria were isolated.

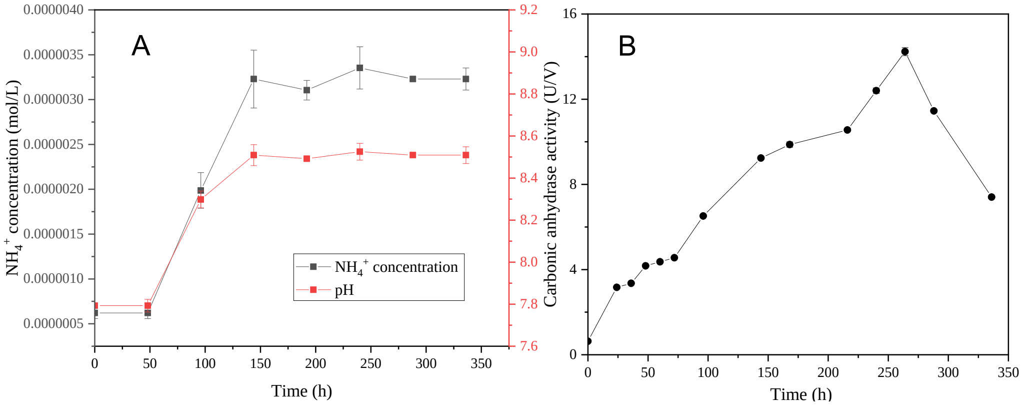

Figure 2 (A) Changes through time of the NH_4^+ concentration and the pH values deduced from the NH_4^+concentration; **(B)** Changes of CA activity.

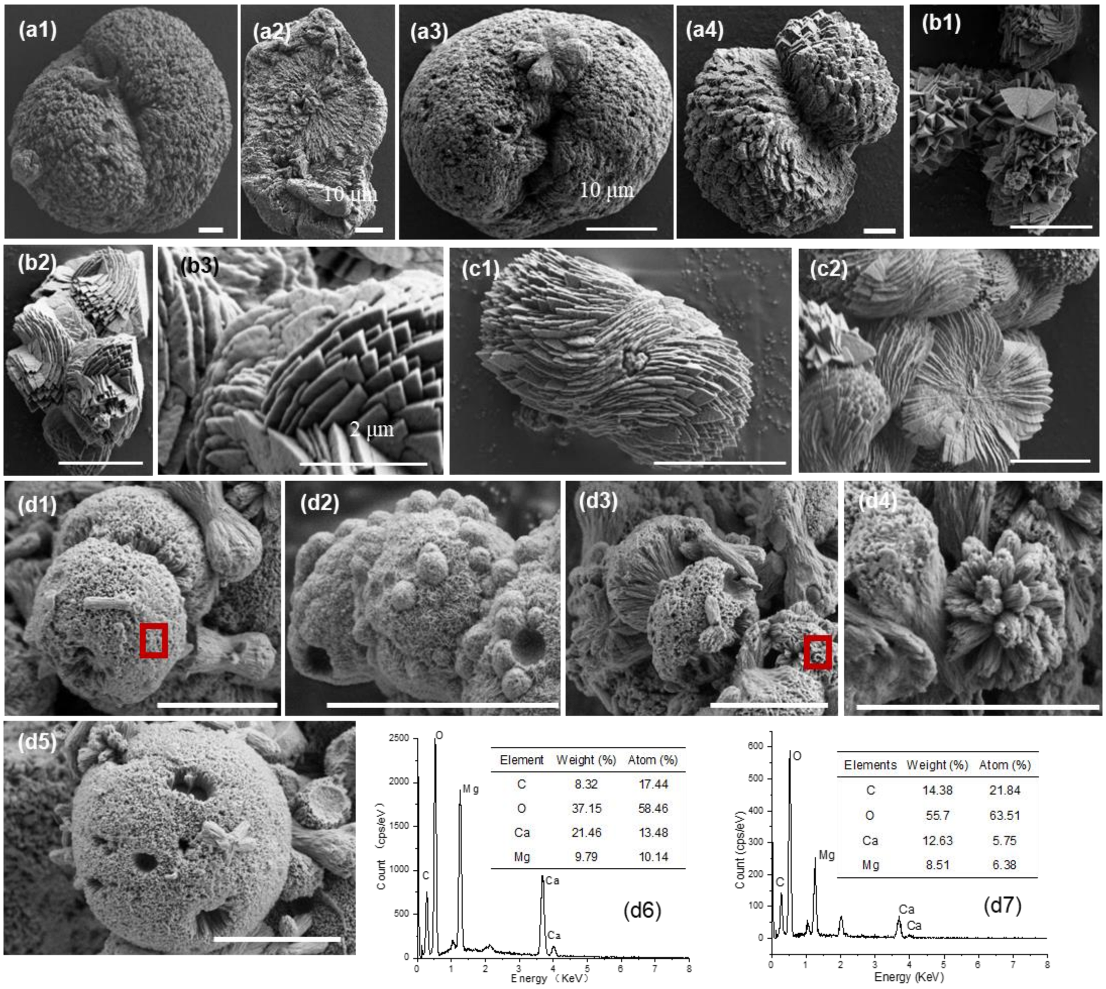

Figure 3 The morphology of minerals induced by *Vibrio harveyi* QPL2 cultured in a medium with Mg/Ca molar ratios of 0, 3, 6, and 10, respectively. **(a 1-4) (b 1-3) (c 1-2) (d 1-5)** correspond to Mg/Ca ratios of 0, 3, 6, and 10, respectively. The unspecified scale is 5 μm. The EDS analysis corresponds to the area of the red box in (d1) and (d3).

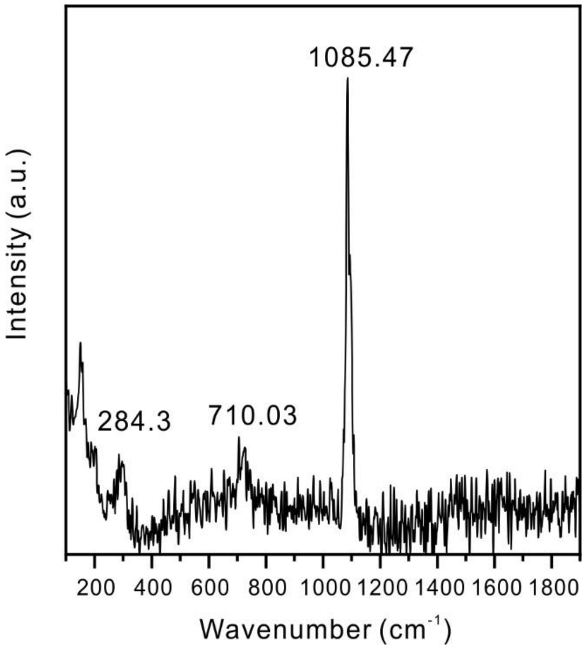

Figure 4 Raman spectra of the protodolomite induced by QPL2 in a medium with a Mg/Ca molar ratio of 12. The peak at 1085.47 cm-1 corresponds to the symmetric CO_3^{2-} vibrational mode (v1). Antisymmetric (v3) and external lattice translational modes are located at 710.03 cm^{-1} and 284.3 cm^{-1}.

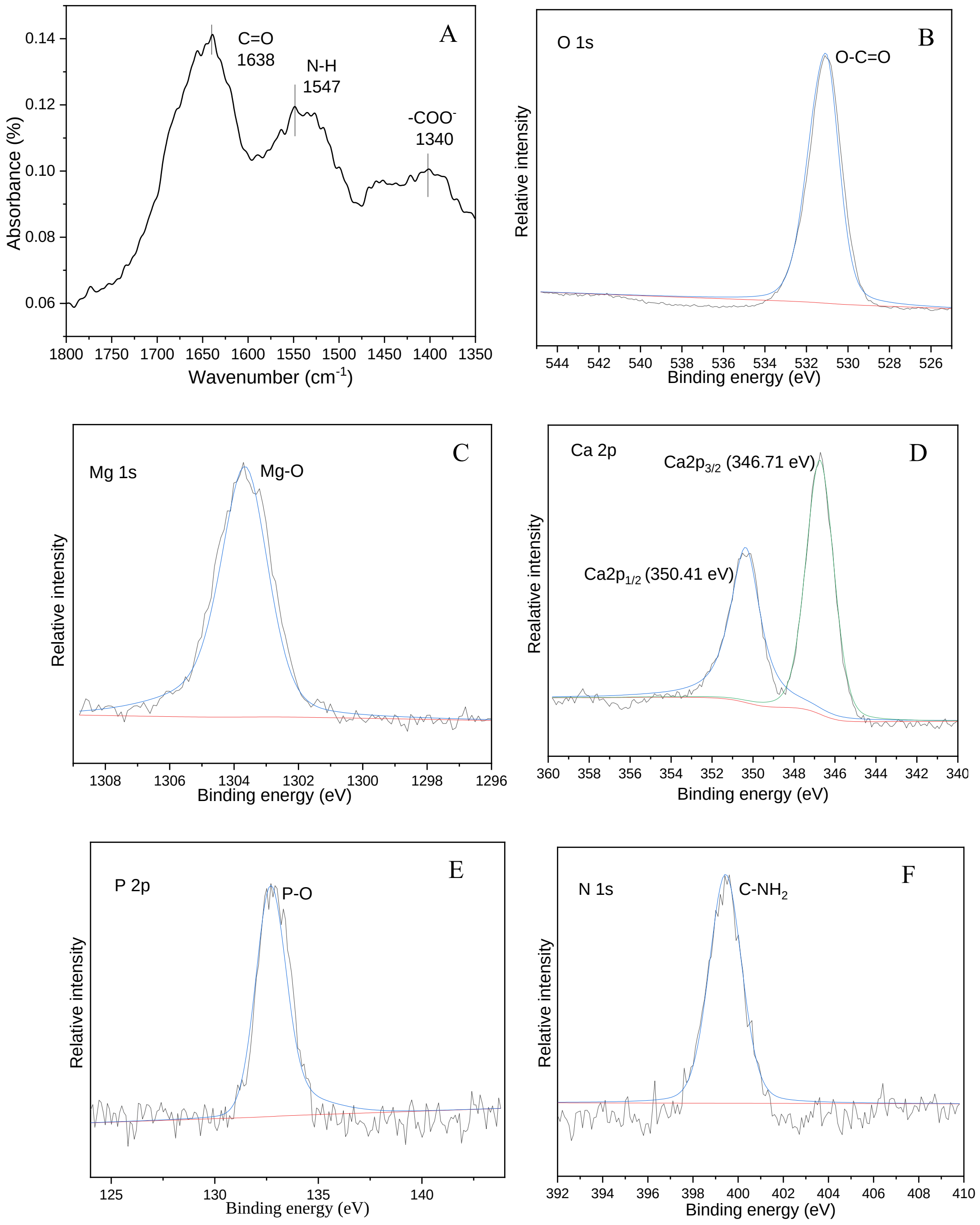

Figure 5 FTIR and XPS analyses. **(A)** The FTIR analysis of EPS isolated from *Vibrio harveyi* QPL2; **(B-F)** XPS analyses of protodolomite induced by *Vibrio harveyi* QPL2.

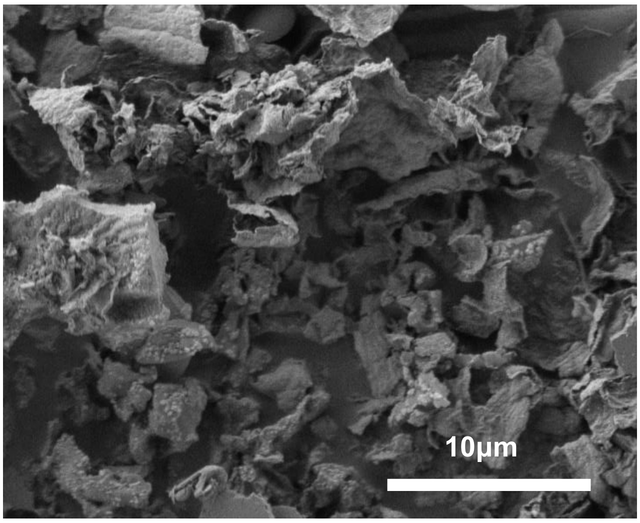

Figure 6 The freeze-dried pure EPS extracted from *Vibrio harveyi* QPL2.

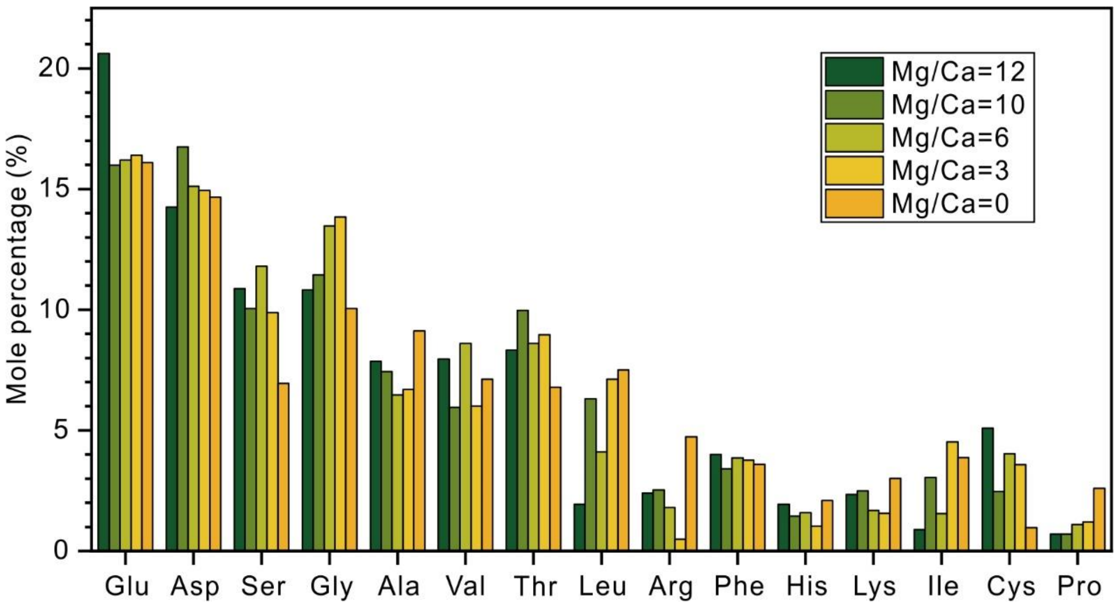

Figure 7 Amino acids in EPS extracted from *Vibrio harveyi* QPL cultured in a medium with Mg/Ca molar ratios of 0, 3, 6, 10, and 12.

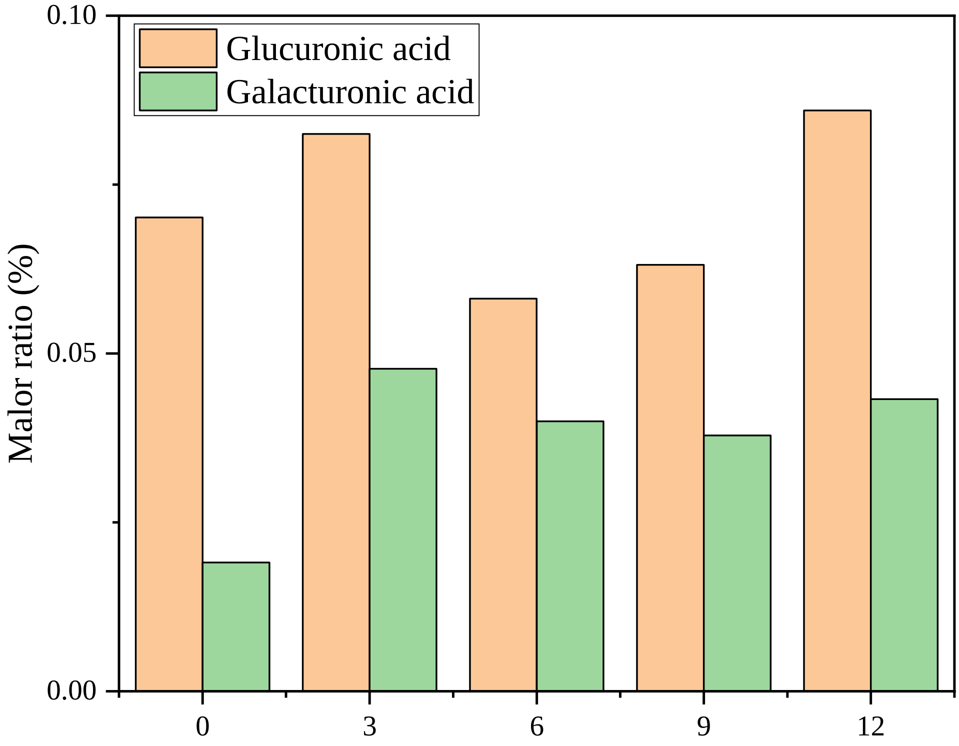

Figure 8 Changes of polysaccharide content under different Mg/Ca ratios.
